# Antivenomics and *in vivo* preclinical efficacy of six Latin American antivenoms towards south-western Colombian *Bothrops asper* lineage venoms

**DOI:** 10.1371/journal.pntd.0009073

**Published:** 2021-02-01

**Authors:** Diana Mora-Obando, Davinia Pla, Bruno Lomonte, Jimmy Alexander Guerrero-Vargas, Santiago Ayerbe, Juan J. Calvete

**Affiliations:** 1 Evolutionary and Translational Venomics Laboratory, Instituto de Biomedicina de Valencia, Consejo Superior de Investigaciones Científicas (CSIC), Valencia, Spain; 2 Instituto Clodomiro Picado, Facultad de Microbiología, Universidad de Costa Rica, San José, Costa Rica; 3 Facultad de Ciencias Naturales, Exactas y de la Educación, Departamento de Biología, Centro de Investigaciones Biomédicas-Bioterio, Grupo de Investigaciones Herpetológicas y Toxinológicas, Universidad del Cauca, Popayán-Colombia; Monash University, AUSTRALIA

## Abstract

**Background:**

*Bothrops asper* represents the clinically most important snake species in Central America and Northern South America, where it is responsible for an estimated 50–80% of snakebites. Compositional variability among the venom proteomes of *B*. *asper* lineages across its wide range mirrors clinical differences in their envenomings. Bothropic antivenoms generated in a number of Latin American countries commonly exhibit a certain degree of paraspecific effectiveness in the neutralization of congeneric venoms. Defining the phylogeographic boundaries of an antivenom's effectivity has implications for optimizing its clinical use. However, the molecular bases and impact of venom compositions on the immune recognition and neutralization of the toxic activities of across geographically disparate populations of *B*. *asper* lineages has not been comprehensively studied.

**Methodology/Principal findings:**

Third-generation antivenomics was applied to quantify the cross-immunorecognizing capacity against the individual components of venoms of three *B*. *asper* lineages (*B*. *asper* (*sensu stricto*), *B*. *ayerbei* and *B*. *rhombeatus*) distributed in south-western (SW) Colombia, of six Latin American antivenoms, produced against homologous (Colombia, INS-COL and PROBIOL) and Costa Rica (ICP)), and heterologous (Argentina (BIOL), Perú (INS-PERU) and Venezuela (UCV)) bothropic venoms. *In vivo* neutralization assays of the lethal, hemorrhagic, coagulant, defibrinogenating, myotoxic, edematogenic, indirect hemolytic, and proteolytic activities of the three SW Colombian *B*. *asper* lineage venoms were carried to compare the preclinical efficacy of three (Colombian INS-COL and PROBIOL, and Costa Rican ICP) antivenoms frequently used in Colombia. Antivenomics showed that all the six antivenom affinity matrices efficiently immunoretained most of the *B*. *asper* lineages venom proteins and exhibited impaired binding towards the venoms' peptidomes. The neutralization profile of the INS-COL, PROBIOL and ICP antivenoms towards the biological activities of the venoms of SW Colombian *B*. *asper* (*sensu stricto*), *B*. *ayerbei* and *B*. *rhombeatus* lineages was coherent with the antivenomics outcome. In addition, the combination of *in vitro* (antivenomics) and *in vivo* neutralization results allowed us to determine their toxin-specific and venom neutralizing antibody content. Noteworthy, heterologous INS-PERU, BIOL, and UCV bothropic antivenoms had equal or higher binding capacity towards the venoms components of SW Colombian *B*. *asper* lineages that the homologous Colombian and Costa Rican antivenoms.

**Conclusions/Significance:**

The combined *in vitro* and *in vivo* preclinical outcome showed that antivenoms manufactured in Colombia and Costa Rica effectively neutralize the major toxic activities of SW Colombian *B*. *asper* lineage venoms. The antivenomics profiles of the heterologous antivenoms manufactured in Argentina, Venezuela, and Perú strongly suggests their (pre)clinical adequacy for the treatment of *B*. *asper* lineage envenomings in SW Colombia. However, their recommendation in the clinical setting is pending on *in vivo* neutralization testing and clinical testing in humans. *Bothrops asper* is a highly adaptable snake species complex, which is considered the most dangerous snake throughout much of its distribution range from the Atlantic lowland of eastern México to northwestern Perú. Antivenoms are the only scientifically validated treatment of snakebite envenomings. Venom variation is particularly common in wide ranging species, such as *B*. *asper*, and may result in variable clinical presentations of envenomings, as is the case for the *B*. *asper* species complex, potentially undermining the efficacy of snakebite treatments depending on the immunization mixture used in the generation of the antivenom. Conversely, phylogenetic conservation of antigenic determinants confers an unpredictable degree of paraspecificity to homologous antivenoms produced for a geographic area, but also to heterologous congeneric antivenoms, towards the venom components of allopatric conspecific populations. This work aimed at comparing the preclinical profile of a panel of Latin American homologous and heterologous antivenoms against the venoms of *B*. *asper* lineages distributed in SW Colombia. The outcome of this study strongly suggests the suitability of considering the heterologous antivenoms BIOL (Argentina), UCV (Venezuela) and INS-PERU (Perú) as alternatives to homologous Colombian INS-COL and PROBIOL and Costa Rican ICP antivenoms for the treatment of envenomings by *B*. *asper* (*sensu stricto*) in W Colombia and Ecuador, *B*. *ayerbei* in Cauca and Nariño (Colombia), and *B*. *rhombeatus* in Cauca river valley, SW Colombia.

## Author summary

Snakebite envenoming is an important occupational health problem, particularly in rural areas of developing countries. The timely administration of an effective antivenom remains the mainstay of snakebite management. However, the use of antivenoms is often limited by non-availability due to high cost or by lack of effectiveness. Antivenom shortage can be addressed through the generation of novel polyspecific antivenoms of wide clinical efficacy against the venoms of the medically-relevant snake species within the geographical range where these antivenoms are intended to be deployed, but also by optimizing the paraspecific use of current antivenoms. In Colombia, antivenoms are supplied by two manufacturers, one public, the Instituto Nacional de Salud (INS), and one private, Laboratorios Probiol (PROBIOL). However, the antivenom supply in Colombia has traditionally been insufficient, a circumstance that has led the Colombian Ministerio de Salud y Protección Social to issue several resolutions and decrees to announce this health emergency in the country, and to import antivenoms produced in México and Costa Rica. Contrary to these countries, where *B*. *asper* represents the only species of the genus, in SW Colombia three close phylogenetically related *B*. *asper* lineages, *B*. *asper* (*sensu stricto*), *B*. *rhombeatus*, and *B*. *ayerbei*, are responsible for most severe cases of snakebite accidents and exhibit remarkable differences in the physiopathological profile of their envenomings. This work aimed to assess the immunorecognition characteristics of a panel of antivenoms manufactured in Colombia, Costa Rica, Argentina, Perú and Venezuela towards the venoms of the three SW Colombian *B*. *asper* lineages. Additionally, combined quantitative *in vitro* and *in vivo* data show that the homologous antivenoms produced in Colombia (INS-COL, PROBIOL) and Costa Rica (ICP) effectively neutralize the lethality and the major toxic activities tested of the three SW Colombian *B*. *asper* lineage venoms. Heterologous Argentinian (BIOL), Venezuelan (UCV) and Peruvian (INS-PERU) antivenoms also showed comparable, even higher, effective immunocapturing ability towards the venom proteomes of SW Colombian *B*. *asper* (*sensu stricto*), *B*. *rhombeatus*, and *B*. *ayerbei*, than the Colombian and Costa Rican antivenoms. These results are in line with previous studies highlighting the notable conservation of paraspecific antigenic determinants across the phylogeny of genus *Bothrops*, and advocate for considering the heterologous Argentinian, Venezuelan and Peruvian antivenoms as further therapeutic alternatives for the treatment of *B*. *asper* spp. snakebites in Colombia.

## Introduction

Snakebite envenoming is an occupational hazard and a WHO category A neglected tropical disease (NTD) [1] that annually kills 81,000–138,000 people living in economically depressed rural communities of Africa, Asia and Latin America, where the provision of health services is limited or inexistent [2,3]. Snakebite leaves victims with permanent physical sequelae and chronic mental morbidity that affects not only the surviving victims, often young agricultural workers but also their entire families, which enter a cycle of generational poverty that is difficult to break [4–6].

In Latin America and the Caribbean 137,000–150,000 snakebite envenomings occur each year, resulting in 3,400–5,000 deaths [2]. With 40,820–44,230 snakebite accidents a year and a mortality rate of 0.05–0.5% [7], the South American subcontinent stands as the most affected region of the New World. Bothropic envenoming is caused by snake species of genera *Bothrops*, *Bothriechis*, *Bothrocophias* and *Porthidium*. Among them, *Bothrops* species have the highest epidemiological importance. Particularly *B*. *asper*, a species complex widely distributed from México to Perú, where it is commonly known as nauyaca, barba amarilla, terciopelo, equis/talla equis, equis patiana, cacica, pelo de gato, boquidorá, mapaná, damá, tapa, etc. (http://snakedatabase.org/species/Bothrops/asper) [8–10], is responsible for 50–80% of the snakebites and 60–90% of fatalities over much of its range [10,11]. It inhabits the tropical rainforest and tropical evergreen forest more frequently, but due to its ease of adapting to different environments it is also found close to crops or human dwellings [12]. *B*. *asper* has crepuscular and nocturnal habits and is recognized as an extremely dangerous snake by its reputation as an irritable snake of large size, rapid reactions and unpredictable behavior, and the high quantity of highly lethal venom (LD_50_ between 2.06 (1.89–2.04) μg newborns' venom/g mouse body weight and 3.82 (3.65–4.00) μg adults' venom/g mouse; doses reported for snakes from Pacific Costa Rican population) that can deliver in a bite [12,13]. Envenomings by *B*. *asper* are characterized by local effects such as edema detectable in the first minutes after the bite; local hemorrhage evident as bleeding or ecchymosis; blisters, regional lymphadenitis, paresthesia, hypothermia, compartment syndrome, dermonecrosis, myonecrosis, abscesses, and gangrene [10,11]. Frequent systemic effects include defibrinogenation, thrombocytopenia, hypotension, massive pulmonary and/or mesenteric capillary micro thrombosis, and these clinical manifestations may become life-threatening as the result of shock and multi-organ failure [10,11].

**Fig 1:**
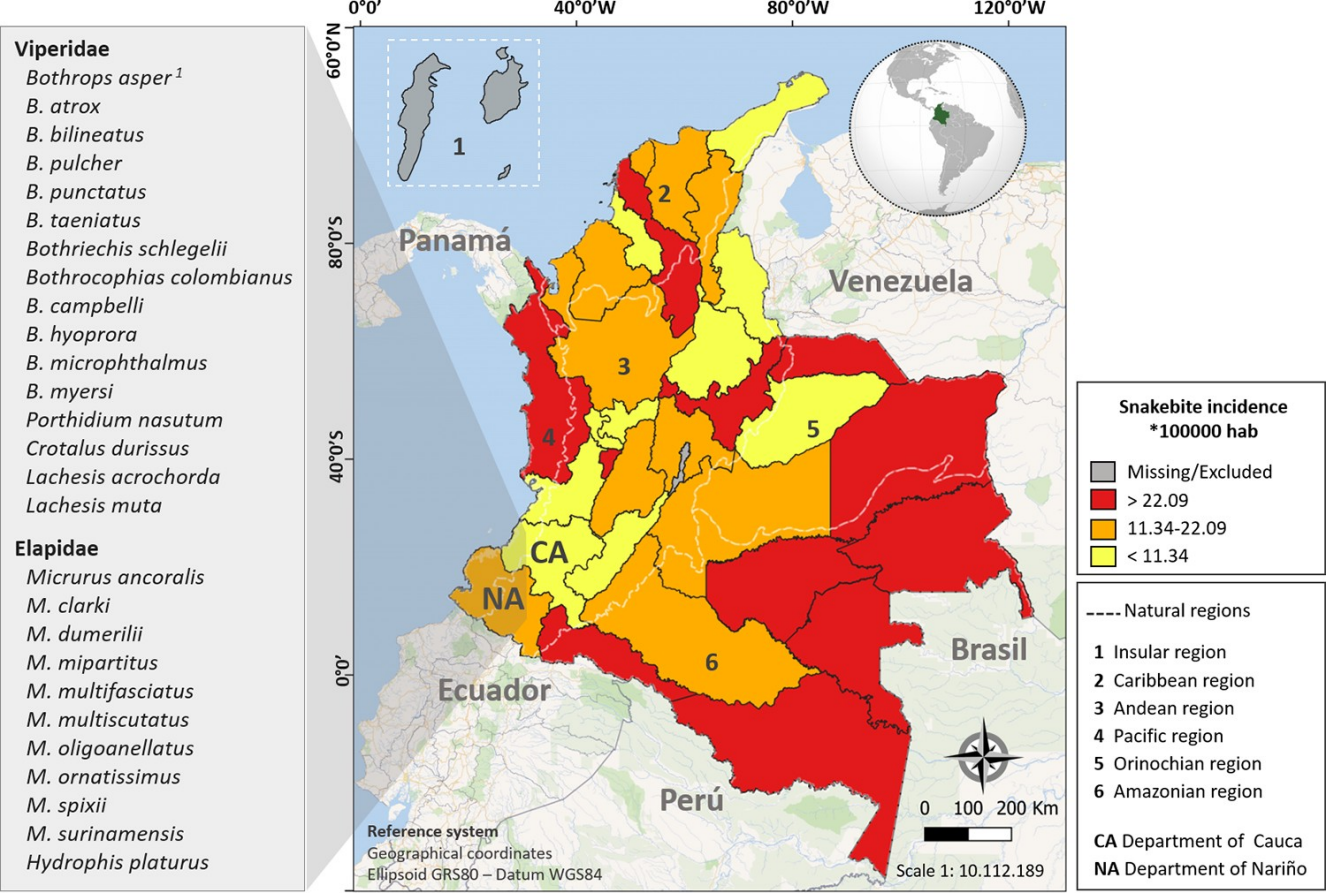
Snakebite incidence in Colombia. The figure is adapted from the event report, epidemiological period XIII, Colombia, 2019 [17]. White dashed lines delimit the Colombian Insular (1), Caribbean (2), Andean (3), Pacific (4), Orinochian (5) and Amazonian (6) natural regions. Snakebite incidence data from the Insular region (San Andrés, Providencia, and Santa Catalina) were "missing or excluded" in the 2019 SIVIGILA-INS event report. The left panel highlights species within the clinically important snake families Viperidae and Elapidae with distribution in the Departments of Nariño and/or Cauca, which can potentially cause snakebite accidents in this region. The list was compiled from Ayerbe and Latorre [22] and Sevilla-Sánchez *et al*. [21]. ^1^*B*. *asper* includes the lineages *B*. *asper* (*sensu stricto*), *B*. *rhombeatus* and *B*. *ayerbei*. The map was prepared in the QGIS software, version 3.14.15-Pi, using public domain maps available in QGIS OpenLayers Plugin, and the Geoportals of the Instituto Geográfico Agustín Codazzi-IGAC (https://geoportal.igac.gov.co/contenido/datos-abiertos-cartografia-y-geografia) and the Departamento Administrativo Nacional de Estadística-DANE (https://geoportal.dane.gov.co/servicios/descarga-y-metadatos/descarga-mgn-marco-geoestadistico-nacional/) from Colombia. All maps were used under a CC-BY 4.0 license.

In Colombia, along the period 2008–2019, the annual cases of snakebites reported by the National Public Health Surveillance System (SIVIGILA) of the National Health Institute (INS) were 3,129 to 5,603 (7–11.1 cases/100,000 population per year) [14–17]. The majority of snakebite envenomings (90–95%) are inflicted by pitvipers of the Viperidae family and involve species from the genera *Bothrops* (*Bothrops asper*, *B*. *atrox*, *B*. *bilineatus*, *B*. *pulcher*, *B*. *punctatus*, *B*. *taeniatus)*, *Bothriechis* (*B*. *schlegelii*), *Bothrocophias* (*B*. *campbelli*, *B*. *colombianus*, *B*. *hyoprora*, *B*. *microphthalmus*, *B*. *myersi*) and *Porthidium* (*P*. *lansbergii*, *P*. *nasutum*), most notably (50–80%) by *B*. *asper* in lowlands and inter-Andean valleys. Two percent of snakebites is due to the bushmaster *Lachesis* spp. (*L*. *acrochorda* and *L*. *muta*) in tropical rain forest; and 1% of envenomings are due to bites by the rattlesnake *Crotalus durissus cumanensis*, a species inhabiting desertic dry or semidry lowlands in the Caribbean region, in the high valley of the Magdalena River, in the Orinochian region, and savannahs (Yarí River) of the Caquetá Department. Ophidian accidents caused by coral snakes (e.g., *M*. *ancoralis*, *M*. *clarki*, *M*. *dissoleucus*, *M*. *dumerilii*, *M*. *mipartitus*, *M*. *nigrocinctus*, *M*. *lemniscatus*, *Micrurus surinamensis*) account for about 1% of the total snakebite envenomings in Colombia. The remaining 1–5% are inflicted by other aglyphous and opisthoglyphous snake species, mainly from the Colubridae family [10,18]. The estimated fatality rate is 1–3% [11,18], although with a pattern of notorious regional variation, and 6–10% of patients suffer some type of life-long sequelae, mainly as a result of dermonecrosis and myonecrosis. Due to their low population density and abundant ophidian fauna, the highest snakebite incidence occurs in the Orinochian and Amazonian regions (Fig 1) [15–17,19], where *B*. *atrox* inflicts 90% of the snakebites [20]

The SW Colombian Departments of Nariño and Cauca are characterized by their impressive mountainous relief belonging to the northern portion of the great Andean mountain system, which extends along the Pacific coast of South America. Its orogeny makes the Colombian Andean natural region one of the most biodiverse in the country, including venomous snakes of the Viperidae and Elapidae families (Fig 1) [21,22]. Saldarriaga-Córdoba *et al*. [23] and Salazar-Valenzuela *et al*. [24] have recently described the existence of phylogeographic structure across *B*. *asper* distribution. Three *B*. *asper* lineages found in Nariño and Cauca Departments (Colombia), *B*. *asper* (*sensu stricto*) on the Pacific versant of the western mountain range, *B*. *ayerbei* in the upper Patia river valley and *B*. *rhombeatus* in the Cauca river valley [25], account for the largest proportion of snakebites in SW Colombia [22]. Two hundred and thirty-nine snakebite cases occurred in Nariño and Cauca in 2019, 56 cases more than 2018. Fig 1 lists the species that potentially cause these accidents in the Departments of Nariño and Cauca. Although this figure is lower than those documented in other Departments (e.g. Antioquia and Norte de Santander) [14–17], the marginal areas where accidents occur along with the limited distribution of antivenoms, among others factors, prevent victims from accessing proper treatment and, as a consequence, there is high underreporting of cases, deaths increase, and physical, psychological, social and economic sequelae are serious [3,21].

The Instituto Nacional de Salud (INS) and Laboratorios Probiol (PROBIOL) manufacture and commercialize in Colombia antivenoms generated from plasma of hyperimmunized horses with mixtures of venoms from *B*. *asper* (*sensu lato*), *B*. *atrox* and *Crotalus durissus cumanensis*, and Laboratorios Probiol additionally includes venom of *Lachesis muta* in the immunization mixture [26]. However, antivenom supply in the country has traditionally been insufficient and the Colombian Ministerio de Salud y Protección Social has issued several resolutions and decrees declaring health emergencies owing to the scarcity of antivenoms, having been necessary to import them from other countries, particularly México and Costa Rica [11,27]. An analysis of the availability of antivenom in the period 1992 to 2016 revealed that compared to the need for +30,000 vials per year, the average national production in that period was 22,000 vials; production exceeded the demand only in some years [27]. Despite antivenom shortage is clearly related to an increase in the mortality rate, and the impact of snakebites could be reduced by the Government demonstrating more interest in this public health issue, initiatives such as the creation of additional antivenom-producing companies have not been materialized [26,27]. In addition, to complicate the picture, clinical observations over two decades have evidenced distinctive local and systemic signs and symptoms of envenoming caused by different *B*. *asper* lineages [28–31], with *B*. *asper* (*sensu stricto*) venom causing stronger myotoxic activity than those of *B*. *ayerbei* and *B*. *rhombeatus*, with the latter venom being the most coagulant, whereas *B*. *ayerbei* venom is poorly myotoxic but the most hemorrhagic among the three lineage venoms [28,29]. In line with these functional data, comparative venomics of *B*. *asper* lineages distributed from México to Ecuador, including venoms from México, Costa Rica, and populations from the Pacific side of Ecuador and SW Colombia, revealed similar overall toxin family compositions albeit exhibiting quantitative differences chiefly in the relative abundance of their four major protein families, snake venom metalloproteinase (SVMP), serine proteinase (SVSP), phospholipase A_2_ (PLA_2_) and C-type lectin-like (CTL) [32].

The cross-reactivity of mono- and polyvalent antivenoms manufactured in México (Birmex, Bioclon), Costa Rica (ICP), Venezuela (UCV), and Colombia (INS-COL, PROBIOL) towards *B*. *asper* venoms from México [33], Guatemala [34], Costa Rica [35,36], Panamá [37], northern Colombia [38], and Ecuador [39] have been reported. The aim of this work was to perform a detailed comparative preclinical analysis of a panel of six antivenoms manufactured in Colombia, Venezuela, Costa Rica, Perú and Argentina to quantify their capability to immunorecognize the toxins of *B*. *asper* lineage venoms (*B*. *asper* (*sensu stricto*), *B*. *rhombeatus*, and *B*. *ayerbei*) from Department of Cauca (Colombia). The neutralization of the lethal and major toxic activities of these *B*. *asper* lineage venoms by the polyvalent Colombian (INS-COL, PROBIOL) and Costa Rican (ICP) antivenoms was also assessed through combined *in vivo* and *in vitro* assays.

## Materials and methods

### Ethics statement

Assays performed in mice were approved by the Institutional Committee for the Care and Use of Laboratory Animals (CICUA) of the University of Costa Rica (approval number 082–08).

### Snake venoms

Venoms of adult snakes from Department of Cauca (Colombia), two of *B*. *asper* from the municipalities of Playa Rica and Huisitó (El Tambo in the Pacific coast), four of *B*. *ayerbei* from the Alto Patía river valley (El Tambo, San Joaquín, Pomorroso, Cauca) and four of *B*. *rhombeatus* from Cauca river valley (Popayán and Cajibío municipalities), were obtained from wild-caught specimens maintained in the serpentarium of the Centro de Investigaciones Biomédicas de la Universidad del Cauca-Bioterio (CIBUC-Bioterio). Venoms were collected by allowing the snake to bite on a glass conical funnel covered with Parafilm. Crude venom was lyophilized and stored at -20°C until used. Equal quantities of each lineage venom were pooled to perform the *in vitro* and *in vivo* assays.

### Antivenoms

Six commercial polyvalent antivenoms (Table 1) were tested: INS-COL from the Instituto Nacional de Salud of Colombia, batch numbers 15SAP01 and 16SAP01 with expiry dates 12/2018 and 04/2019, respectively; PROBIOL produced by Laboratorios Probiol S.A. (Colombia), batch number AP066XI16-ES with expiry date 11/2018; ICP-polyvalent from Instituto Clodomiro Picado (Costa Rica), batch 6060618 POLF with expiry date 12/2023; INS-PERU from Instituto Nacional de Salud of Perú, batch 10200045 with expiry date 02/2018; UCV, produced by Centro de Biotecnología de la Unidad de Farmacia of the Universidad Central de Venezuela, batch 179 with expiry date 07/2017; and BIOL produced by Instituto Biológico Argentino S.A.I.C., batch 3664 with expiry date 10/2018.

**Table 1:** Characteristics of the equine polyvalent antivenoms used in this study.

Manufacturer (Abbreviation)	Active substance^a^	State	Color	Excipient (odor)	pH	Protein [mg/mL]	Neutralizing potency per vial (mg venom/10 mL antivenom)^a^
Instituto Nacional de Salud, Colombia (INS-COL)	IgG	Liquid	None	Weak	6	56.8 ± 3.2^b^	*Bothrops sp* (70 mg), *Crotalus sp* (10 mg).
Laboratorios Probiol S.A, Colombia (PROBIOL)	IgG	Lyophilized	Greenish yellow	Strong	7	200.7^b^	*Bothrops asper* (25 mg), *B*. *atrox* (25 mg), *Lachesis muta* (10 mg), *Crotalus durissus* (5 mg).
Instituto Clodomiro Picado, Costa Rica (ICP)	IgG	Lyophilized	Light Blue	Strong	6	59.7 ± 0.1^b^	*Bothrops asper* (30 mg), *Crotalus simus*, *Crotalus vegrandis* (20 mg), *Lachesis stenophrys* (30 mg).
Instituto Nacional de Salud, Perú (INS-PERU)	IgG	Liquid	None	Weak	6	59.1 ± 0.1^b^	*Bothrops atrox*, *B*. *brazili*, *B*. *pictus*, *B*. *barnetti*, *Bothrocophias hyoprora* (25 mg).
Centro de Biotecnología de la Unidad de Farmacia de la Universidad Central de Venezuela (UCV)	F(ab')_2_	Liquid	None	N.D	N.D	41.9^c^	*Bothrops colombiensis* (20 mg), *Crotalus durissuss cumanensis* (15 mg).
Instituto Biológico Argentino S.A.I.C (BIOL)	F(ab')_2_	Lyophilized	White	N.D	N.D	59.3^b^	*Bothrops alternatus* (12.5 mg), *B*. *diporus* (12.5 mg), *Crotalus durissus* (4 mg).

^a^ Information obtained from the inserts of the products.

^b^ Protein concentration was estimated by Lambert-Beer Law.

^c^ Data provided by the manufacturer (Mariana del Valle Cepeda Briceño, personal communication). N.D: not determined.

All antivenoms were generated from plasma of horses hyperimmunized against different mixtures of venoms: *B*. *atrox* (Meta and Casanare, Orinoquía; Amazonas and Caquetá, Amazonía), *B*. *asper* (*sensu stricto*, Departments of Atlántico, Bolívar, Cauca (Isla Gorgona), Cauca river valley (Pacific region), and Tolima); *B*. *rhombeatus*, Antioquia), and *Crotalus*. *d*. *cumanensis* (INS-COL); *B*. *atrox* (Meta and Casanare, Orinoquia; Amazonas and Caquetá, Amazonía), *B*. *asper* (*sensu stricto*, Costa Atlántica; *B*. *rhombeatus*, Magdalena Medio river valley and Cauca river valley), *Crotalus*. *d*. *cumanensis*, and *Lachesis muta* (PROBIOL); *B*. *asper*, *C*. *simus*, *Crotalus vegrandis* and *L*. *stenophrys* (ICP); *B*. *atrox*, *B*. *barnetti*, *B*. *brazili*, *B*. *pictus*, and *Bothrocophias hyoprora* (INS-PERU); *B*. *atrox*, *B*. *colombiensis*, *B*. *venezuelensis*, *Porthidium lansbergii hutmanni*; *C*.*d*. *cumanensis*, and *C*.*d*. *ruruima* (UCV); and *B*. *asper*, *C*. *simus*, and *L*. *muta* (BIOL). INS-COL, PROBIOL, ICP, INS-PERU antivenoms are composed of whole immunoglobulins (IgGs), whereas the UCV and BIOL antivenoms are made up of the antigen-binding fragments F(ab')_2_ purified from pepsin-digested whole hyperimmune serum.

### Characterization of the antivenoms

Physicochemical characteristics of the antivenoms, such as color, odor, and pH were examined and recorded. The antivenom total protein concentration (mg/mL) was determined spectrophotometrically using an extinction coefficient for a 1 mg/mL concentration (ε^0.1%^) at 280 nm of 1.36 (mg/mL)^−1^ cm^−1^ [40,41]. The homogeneity and purity of the antivenoms were assessed by non-reducing SDS-PAGE analysis in 8.5% polyacrylamide gels and proteomics characterization of the Coomassie Brilliant Blue G-250-stained protein bands [42]. To this end, protein bands of interest were excised, subjected to in-gel reduction (10 mM dithiothreitol, 30 min at 65°C) and alkylation (50 mM iodacetamide, 2 h in the dark at room temperature), followed by overnight digestion with sequencing-grade trypsin (66 ng/μL in 25 mM ammonium bicarbonate, 10% ACN; 0.25 μg/sample) using an automated Genomics Solution ProGest Protein Digestion Workstation. Tryptic digests were dried in a vacuum centrifuge (SPD SpeedVac, ThermoSavant), redissolved in 15 μL of 5% ACN containing 0.1% formic acid, and submitted to LC-MS/MS. Tryptic peptides were separated by nano-Acquity UltraPerformance LC (UPLC) using BEH130 C_18_ (100μm × 100 mm, 1.7μm particle size) column in-line with a Waters SYNAPT G2 High Definition Mass Spectrometry System. The flow rate was set to 0.6 μL/min with a linear gradient of 0.1% formic acid in MilliQ water (solution A) and 0.1% formic acid in ACN (solution B) with the following conditions: isocratically 1% B for 1 min, followed by 1–12% B for 1 min, 12–40% B for 15 min, 40–85% B for 2 min. For CID-MS/MS, the electrospray ionization source was operated in positive ion mode and both singly- and multiply-charged ions were selected for CID-MS/MS at sample cone voltage of 28 V and source temperature of 100°C. The LC–MS eluate was continuously scanned from 300 to 1990 m/z in 1 sec and peptide ion MS/MS analysis was performed over the range m/z 50–2000 with scan time of 0.6 sec. Fragmentation spectra were i) match against the last update of the NCBI non-redundant database (release 239.0 of 8/18/2020) using the on-line form of the MASCOT Server (version 2.6) at http://www.matrixscience.com, and ii) processed in Waters Corporation's ProteinLynx Global SERVER 2013 version 2.5.2. (with Expression version 2.0). The following search parameters were used: Taxonomy: all entries; enzyme: trypsin (2 missed cleavage allowed); MS/MS mass tolerance was set to ± 0.6 Da; carbamidomethyl cysteine and oxidation of methionine were selected as fixed and variable modifications, respectively. The relative abundances (% of total protein bands area) of antivenom components were estimated by densitometry of Coomassie-stained SDS-polyacrylamide gels using Image Studio Lite, version 5.2 (LI-COR Biosciences) software, and the relative abundances of different proteins contained in the same SDS-PAGE bands were estimated based on the relative ion intensities of the most abundant MS/MS-derived tryptic peptide ions associated with each protein.

### Third-generation antivenomics

Third-generation antivenomics was applied to assess the immunorecognition ability of the antivenom [43,44]. Antivenoms (INS-COL, PROBIOL, ICP, INS-PERU, UCV, BIOL) were dialyzed against distilled water, lyophilized, and reconstituted in coupling buffer (0.2 M NaHCO_3_, 0.5 M NaCl, pH 8.3). Affinity columns were prepared in batch as follow: 3 mL of CNBr-activated Sepharose 4B matrix (Ge Healthcare, Buckinghamshire, UK) packed in an ABT column (Agarose Bead Technologies, Torrejón de Ardoz, Madrid), later washed with 10x matrix volumes of cold 1 mM HCl (to preserve the activity of reactive groups) followed by two matrix volumes of coupling buffer to adjust the pH of the column to 7.0–8.0. ~100 mg of each lyophilized antivenom was dissolved in 2x matrix volumes of coupling buffer and incubated with 3 mL CNBr-activated matrix overnight at 4°C. The amount of coupled protein (IgG or F(ab’)_2_) was determined as the difference between the quantity of protein incubated and the quantity uncoupled measured spectrophotometrically at 280 nm before and after incubation with the matrix. After the coupling, any remaining active-matrix groups were blocked with 6 mL of 0.1 M Tris-HCl, pH 8.5 overnight at 4°C and the excess of uncoupled antibody was eliminated by alternatively washing 6 times with 3x matrix volumes of 0.1 M acetate buffer (0.5 M NaCl, pH 4.0–5.0), and 3x matrix volumes of 0.1 M Tris-HCl buffer (0.5 M NaCl, pH 8.5). Five affinity columns each containing 8 mg of immobilized antivenom were equilibrated with three volumes of PBS (20 mM phosphate buffer, 135 mM NaCl, pH 7.4) and incubated with increasing amounts (100–1200 μg of total venom proteins) of venom (*B*. *asper* (*sensu stricto*), *B*. *rhombeatus*, or *B*. *ayerbei*) dissolved in 350 μL of PBS, and the mixtures were incubated for 1 h at room temperature in an orbital shaker. In parallel, as specificity controls, the highest amount of venom was incubated with 350 μL of mock matrix and 350 μL of matrix coupled with 8 mg of naïve equine IgG. The non-retained fractions of columns incubated with 100–300 μg, 600 μg, 900 μg, and 1200 μg were recovered with 2x, 4x, 6x and 8x matrix volumes of PBS, respectively, and the immunocaptured proteins were eluted with 3x (100–300 μg) and 6x (600–1200 μg) matrix volumes of 0.1M glycine-HCl, pH 2.7 buffer and brought to neutral pH with 1M Tris-HCl, pH 9.0. The column fractions (non-retained and retained) with highest volume (600–1200 μg) were divided equally to avoid concentrating or diluting the total proteins too much as follows: fractions recovered in 600 μg, 12; non-retained and retained fractions in 900 μg, 13 and 12; non-retained and retained fractions in 1200 μg, 14 and 12. These aliquots were concentrated in a Savant SpeedVac vacuum centrifuge (ThermoFisher Scientific, Waltham, MA USA) to 40 μL and fractionated by reverse-phase HPLC using an Agilent LC 1260 High-Pressure Gradient System (Santa Clara, CA, USA) equipped with a Discovery BIO Wide Pore C_18_ (15 cm×2.1 mm, 3 μm particle size, 300 Å pore size) column and a DAD detector. Elution was monitored at 215 nm with a reference wavelength of 400 nm [43,44].

The percentage of immunorecognition was obtained by the integration of RP-HPLC profiles of retained and non-retained fractions. The fraction of non-immunocaptured molecules was estimated as the relative ratio of the chromatographic areas of the proteins recovered in the non-retained (NR) and retained (R) affinity chromatography fractions applying the equation %NRi = 100-[(Ri|(Ri+NRi)) x 100], where Ri corresponds to the area of the same protein “i” in the chromatogram of the fraction retained and eluted from the affinity column. This result was corrected for toxins such as SVMP which elute with difficulty from the column due to their high binding affinity to the immobilized antivenom. In this case, the percentage of non-immunocaptured toxin “i” (% NRtoxin“i”) was calculated as the ratio between the chromatographic areas of the same peak recovered in the non-retained fraction (NRtoxin“i”) and in a reference venom (Vtoxin“i”) containing the same amount of total protein that the parent venom sample and run under identical chromatographic and experimental conditions, using the equation %NRtoxin "i" = (NRtoxin "i"/Vtoxin "i") x 100 [45].

### Neutralization of biological effects of venoms

The neutralizing capacity of antivenoms manufactured in Colombia (INS-COL, PROBIOL) and Costa Rica (ICP) towards major bothropic biological effects (i.e., lethality, hemorrhagic, coagulant, myotoxic, defibrinogenating, edematogenic, indirect hemolytic, and proteolytic), determined in this study or previously reported by Mora-Obando *et al*. [28] and Rengifo-Ríos *et al*. [29] for the same venoms, was tested using standardized protocols recommended by the WHO [46–48] with slight modifications. In particular, although WHO recommends "using groups of 5–6 mice (of the same strain and weight range used for the LD_50_ assay) for ED_50_ determinations, although 10 mice may be needed for some venoms". In our experience with bothropoid venoms 4 mice groups and 4–6 levels of antivenom:venom ratios provide strong statistics for calculating ED_50_s with narrow 95% confidence intervals. Thus, to comply with the principles of the 3Rs (Replacement, Reduction and Refinement) and the ARRIVE guidelines (Animal Research: Reporting of *in vivo* Experiments) (NC3Rs, https://www.nc3rs.org.uk/arriveguidelines), without compromising the robustness of the ED_50_ data, here the number of animals was 4 per antivenom:venom level. The median lethal dose (LD_50_) of *Bothrops rhombeatus* venom was determined using Probits [28,49]. All neutralization assays were performed by incubating for 30 min at 37°C constant amounts of venom (challenge dose) (Table 2) with increasing dilutions of antivenoms, previously dialyzed and lyophilized and dissolved to a final concentration of 70 mg/mL in PBS [50]. In each experiment, positive and negative control mice groups were injected, respectively, with venom in PBS and antivenom alone. After each assay, mice were euthanized by CO_2_ inhalation [48].

**Table 2:** Reference doses of *B*. *asper* venoms considered for the experimental design of this study.

Lineage	LD_50_ [μg/mouse]	MHD [μg]	MCD [μg]	MDD [μg]
*B*. *asper* (*sensu stricto*)	100.9 (83.2–122.8)^a^	1.44 ± 0.20^a^	0.37 ± 0.05^a^	2.0^a^
*B*. *rhombeatus*	54.9 (36.0–83.8)^b^	3.55 ± 0.30^c^	0.21 ±0.03^c^	3.0^b^
*B*. *ayerbei*	50.1 (37.4–58.3)^a^	0.24 ± 0.04^a^	0.96 ± 0.10^a^	3.0^a^

LD_50_: Median Lethal Dose; MHD: Minimum Hemorrhagic Dose; MCD: Minimum Coagulant Dose; MDD: Minimum Defibrinogenating Dose. The challenge doses were: (a) lethal activity: four LD_50_; (b) hemorrhagic activity: ten MHD; (c) coagulant activity: two MCD and (d) defibrinating activity: two MDD.

^a^ Mora-Obando *et al*. [28]

^b^ this work

^c^ Rengifo-Ríos *et al*. [29].

#### Neutralization of venom lethality

Groups of four CD-1 mice (16–18 g) received intraperitoneal injections of four LD_50_s (challenge dose) (Table 2) mixed with antivenom in different proportions (250–1000 μL antivenom/mg venom) dissolved in PBS. Forty-eight hours later, the number of surviving mice in each group was recorded. The antivenoms' capacity to neutralize the venoms' lethal activity was expressed as median effective dose (ED_50_), which corresponds to the amount of antivenom that protects 50% of the injected mice [35]. ED_50_ value and its 95% confidence limits for each venom was calculated using Probit analyses with software BioStat v. 2008.

#### Calculation of the venom toxin-specific and venom neutralizing antibody content of antivenoms

The data from the antivenomics and ED_50_ experiments were combined to calculate the fraction of anti-toxin and venom neutralizing IgG or F(ab’)_2_ molecules [41]. Briefly, the percentage of anti-toxin IgG or F(ab’)_2_ molecules in the antivenoms was calculated by dividing [(1/2 maximal amount (in μmoles) of total venom proteins bound per antivenom vial) x molecular mass (in kDa) of antibody (IgG, 160 kDa or F(ab’)_2_, 110 kDa) molecule] by the [total amount of antibody (IgG or F(ab’)_2_) (in mg) per antivenom vial] [44,51,52]. To calculate the percentage of lethality neutralizing antibodies, the potency (P) of the antivenoms was first obtained, i.e. the amount of venom (mg) neutralized per 1 mL of each antivenom, was calculated as P = [(n-1)/ ED_50_]×LD_50_, where “n” is the number of LD_50_s used as challenge dose to determine the ED_50_ and, "n-1" is used because at the endpoint of the neutralization assay, the remaining activity of one LD_50_ remains unneutralized causing the death of 50% of mice [53,54]. For the calculation of P, LD_50_ and ED_50_ values were expressed, respectively, in mg venom/mouse and mL of antivenom that protect 50% of the mice population inoculated with n×LD_50_. Finally, the antivenom's potency (P) was divided by the maximal amount of total venom proteins bound by mL of antivenom [41].

#### Neutralization of hemorrhagic activity

Groups of four mice (18–20 g) received intradermal injections of 0.1 mL each containing 10 Minimum Hemorrhagic Doses (MHDs) of venom (Table 2) incubated with increasing dilutions of antivenom (62.5–1000 μL antivenom/mg venom). Two hours post-injection mice were sacrificed by CO_2_ inhalation, their skin removed, and the area of the hemorrhagic lesion photographed. The images were processed with software Inkscape v. 0.92 as described by Jenkins *et al*. [55]. The antivenom neutralization capacity (MHDED_50_) was defined as the venom/antivenom ratio that reduced the diameter of the hemorrhagic lesion to 50% compared to the diameter of the lesion in the positive control [50].

#### Neutralization of coagulant activity

0.1 mL aliquots each containing a fixed-dose of venom (Table 2) incubated with antivenom at different ratios (7.8–1000 μL antivenom/mg venom) were added to 0.2 mL of citrated human plasma preincubated for 30 min at 37°C. Clotting times were recorded and the Effective Dose (ED) of the antivenom was calculated as the venom/antivenom ratio which prolonged the clotting time three times compared to that of plasma incubated with venom alone [56].

#### Neutralization of defibrinogenating activity

Groups of four CD-1 mice (18–20 g) received intravenous injections of 200 μL of PBS containing two Minimum Defibrinogenating doses (MDDs) (Table 2) incubated with antivenom at different ratios (62.5–2000 μL antivenom/mg venom). One hour post-injection the animals were anesthetized and bled to obtain around 0.2 mL of blood, which was left undisturbed for two hours at room temperature. Neutralization ability of the antivenom was expressed as effective dose (ED), defined as the lowest venom/antivenom ratio in which blood coagulation occurred in all the mice [56].

#### Neutralization of myotoxic activity

A fixed-dose of venom (50 μg) incubated with antivenom at ratios of 250, 500, and 1000 μL antivenom/mg venom were injected in groups of four mice (18–20 g) into the right gastrocnemius muscle. 3 h later, a blood sample was obtained from the caudal vein and the plasma creatine kinase (CK) activity, expressed as units/L, was measured using a UV kinetic assay (CK-Nac, Biocon Diagnostik). The neutralization capacity of the antivenom was expressed as ED_50_, defined as the venom/antivenom ratio where CK levels were reduced by 50% compared to the positive control [48,57].

#### Neutralization of edematogenic activity

Each animal of groups of four mice (18–20 g) received a subcutaneous injection in the right footpad, containing a mixture of 5 μg of venom incubated with antivenoms at final ratios of 250–1000 μL antivenom/mg venom, in a total volume of 50 μL of PBS. The left footpad injected with PBS alone served as negative control. Footpads' thickness, measured using a low-pressure spring caliper (Oditest) 0.5, 1, 3, and 6 h post-injection, was considered a quantitative indicator of edema, and the data were expressed as percentage. The antivenoms' neutralizing capacities were determined at 60 min and the ED_50_ expressed as the venom/antivenom ratio in which edema was reduced by 50% compared to the positive control [58].

#### Neutralization of indirect hemolytic activity

The indirect hemolytic activity of each venom was determined following the method described by Arce-Bejarano *et al*. [59], with slight modifications, using rabbit erythrocytes in a tube fluid phase system. Briefly, rabbit blood was collected by venipuncture in Falcon tubes containing Alsever's solution (2.05% dextrose, 0.8% sodium citrate, 0.055% citric acid, and 0.42% sodium chloride) as anticoagulant, and centrifuged at 400 xg for 5 min. Red blood cells were washed five times in 0.14 M NaCl, 0.01 M Tris, pH 7.7 (TBS). 50 μL of a reaction mixture containing a 5% suspension of erythrocytes in TBS gently mixed with 0.25% w/v *sn*-3-phosphatidylcholine was incubated with 250 μL containing various amounts of venom diluted in TBS-Ca^2+^ (TBS supplemented with 10mMCaCl_2_, pH 7.7) in 96-well plates. Identical mixtures without venom or replacing venom by 0.1% Triton X-100 in water were included as negative (0% hemolysis) and positive (100% hemolysis) controls, respectively. The plates were incubated for 60 min at 37°C with mild stirring every 20 min, centrifuged at 400 xg for 5 min, and the absorbance of the supernatants measured at 540 nm using a microwell plate reader was considered a quantitative index of hemolysis. Experiments were performed in triplicates and the results were expressed as percentages of the hemolysis recorded for the positive control. For assessing an antivenom's neutralization activity, a fixed challenge dose defined as three times the amount of venom that hemolyzed 50% of the erythrocytes was incubated at increasing ratios of antivenom (250–1000 μL antivenom /mg venom) for 30 min at 37°C, after which 50 μL of the 5% suspension of erythrocytes/0.25% w/v *sn*-3-phosphatidylcholine was added, and the indirect hemolytic activity measured as above. The neutralizing capacity of antivenom (ED_50_) was defined as the venom/antivenom ratio in which the venom-induced hemolytic activity was reduced by 50% compared to the positive control [59].

#### Neutralization of proteolytic activity

The proteolytic activity of each venom was determined according to the method described by Gutiérrez *et al*. [50]. Briefly, 20 μL aliquots containing 2.5–40 μg of venom were added to 100 μL of substrate (10 mg/mL azocasein in 25 mM Tris, 150 mM NaCl, 5 mM CaCl_2_, pH 7.4) and incubated at 37°C for 90 min in 96-well plates. Reactions were stopped by adding 200 μL of 5% trichloroacetic acid and centrifuged (350 xg x 5 min). Hundred fifty μL of supernatant were mixed with the same volume of 0.5 M NaOH and the absorbance at 450 nm recorded. For antivenom neutralization testing, the challenge dose corresponded to the amount of venom capable of inducing a change in absorbance of 0.5 at 450 nm. Challenge doses of each venom, either alone or incubated with antivenom at several ratios of (250, 500, and 1000 μL antivenom/mg venom) in a total volume of 25 μL, were tested as above. The ED_50_ antivenom's neutralizing capacity was expressed as the venom/antivenom ratio in which proteolysis was reduced by 50% compared to the positive control [50].

#### Enzyme-linked immunosorbent assay (ELISA)

Antibody titers of the antivenoms were determined by ELISA. For this end, 1 μg samples of venom dissolved in 100 μL of PBS (20 mM phosphate, 135 mM NaCl, pH 7.4), were coated overnight at 4°C per well of 96-well plates (Dynatech Immulon, Alexandria, VA). Thereafter, the plates were decanted and free sites in each venom-coated well was blocked with 100 μl/well of 1% bovine serum albumin (BSA) in PBS for 30 min at room temperature. Antivenoms dissolved in PBS to a concentration of 70 mg/mL were serially diluted (1:1000–1:128000) in PBS containing 1% BSA, added to the wells and incubated for 1 h at room temperature. The plates were then washed five times with PBS and bound antibodies detected following incubation for 1 h at room temperature with 100 μL of a 1:2000 dilution of anti-horse IgG-phosphatase-conjugate (Sigma, St. Louis, MO, USA) in FALC buffer (0.05 M Tris, 0.15 M NaCl, 20 μM ZnCl_2_, 1mM MgCl_2_, pH 7.4) containing 1% BSA. Finally, the plates were washed five times with FALC buffer, the substrate *p*-nitrophenyl phosphate (1mg/ml) in diethanolamine buffer (1mM MgCl_2_, 90mM diethanolamine, pH 9.8) was added, and the absorbance at 405 nm was recorded for 5–60 min using a microplate reader (Multiskan Labsystems Ltd., Helsinki, Finland).

### Statistical analyses

Results were represented as dose-response graphs and reported as the mean ± SD of duplicate, triplicate, or quadruplicate determinations depending on the biological activity assessed. Antivenom ED_50_s against venom lethality was calculated using the software BioStat v. 2008, and the values were considered significantly different among groups when the 95% confidence limits did not overlap. ED_50_s towards venom hemorrhagic, coagulant, edematogenic, proteolytic and myotoxic activities were calculated from the equations of the regression analyses in Excel (Microsoft Office 2019). The significance of the differences between the experimental groups was determined by parametric or non-parametric analysis of variance (ANOVA or Kruskal–Wallis, respectively), followed by *post hoc* tests to identify significant differences between group pairs. Differences with p ≤ 0.05 were considered statistically significant. All the statistical analyses were performed using Software IBM SPSS Statistics version 23.

## Results and discussion

The ability of different mono- and polyvalent experimental and commercial bothropic antivenoms to neutralize the most relevant toxic effects produced by *B*. *asper* bites was demonstrated in the late 1990s [60–62], and the outcome clearly showed that the antivenoms were more effective in preventing the systemic than the local (myotoxic, dermonecrotic and hemorrhagic) tissue damaging effects [63–65]. Current antivenoms manufactured in Colombia are generated from plasma of horses hyperimmunized with mixtures of *B*. *atrox*, *B*. *asper* and *C*.*d*. *cumanensis* venoms supplemented (Laboratorios Probiol) or not (Instituto Nacional de Salud, INS-COL) with venom of the bushmaster *L*. *muta*. Given the wide range of *B*. *asper* and the occurrence of intraspecific phylogeographic structure [23,24,32], it is not surprising [62] that different antivenom effectiveness has been observed regarding the number of vials needed in the treatment of envenomings inflicted by snakes from Pacific region than from the Andean region (S. Ayerbe, personal communication). Therefore, the aim of this study was to determine the immunological characteristics of a panel of bothropic antivenoms that includes the national products as well as those imported or potentially importable antivenoms, which may complement national production in the treatment of envenomings caused by the three lineages of the *B*. *asper* complex distributed in SW Colombia, *B*. *asper* (*sensu stricto*), *B*. *rhombeatus*, and *B*. *ayerbei*, whose structural and functional venomics profiles have recently been reported [28,32].

### Physicochemical characterization of the antivenoms

**Fig 2:**
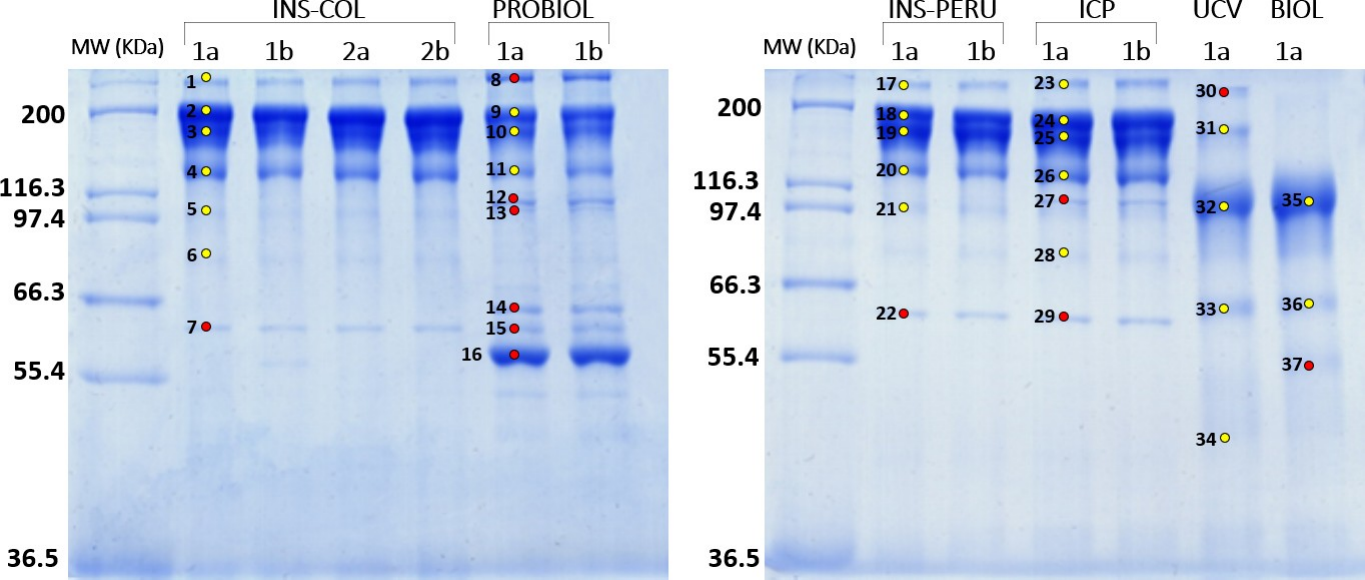
SDS-PAGE analysis of the polyvalent antivenoms used manufactured by Instituto Nacional de Salud (Colombia) (INS-COL), Laboratorios Probiol S.A, (Colombia) (PROBIOL), Instituto Nacional de Salud de Perú (INS-PERU), Instituto Clodomiro Picado (Costa Rica) (ICP), Centro de Biotecnología de la Unidad de Farmacia de la Universidad Central de Venezuela, (UCV), and Instituto Biológico Argentino S.A.I.C. (BIOL). Lanes 1a/b or 2a/b, vials used in the experiments. Molecular weight markers (MW) are indicated on the left. Coomassie Brilliant blue-stained bands labeled 1–37 were excised and submitted to tandem mass spectrometry analysis (S1 Table). Bands containing IgG aggregation or degradation products are identified by yellow-filled circles. Red-filled circles denote non-IgG proteins bands.

Table 1 summarizes the physicochemical characteristics of the 6 antivenoms (INS-COL, PROBIOL, INS-PERU, ICP, UCV, BIOL) examined. Total protein concentration was similar among INS-COL, INS-PERU, ICP and BIOL antivenoms, ranging from 56.8 to 59.7 mg/mL, whereas UCV had lowest (41.9 mg/mL) and PROBIOL presented a much higher (200.7 mg/mL) protein content. SDS-PAGE analysis (Fig 2) confirmed the type of antibody molecule specified in the products' inserts (IgG or F(ab’)_2_) (Table 1), but also revealed a number of other protein bands. Protein spots excised from the SDS-PAGE gels were identified through tandem-mass spectrometry (MS/MS) analysis as aggregation and degradation products of IgG (Fig 2, yellow-filled circles) and non-IgG proteins (Fig 2, red-filled circles) (S1 Table)

The INS-COL antivenom showed a predominance (82.3%) of intact IgG glycoforms (Fig 2, bands 2 and 3) and their aggregation (band 1, 2.9%) and degradation (4, 11.6%; 5, 1.3%; 6, 0.7%) products, and a 1.1% of α_1_B-glycoprotein (Fig 2, band 7). In comparison, PROBIOL antivenom contained significantly lower intact IgG bands (41.8%), comparable IgG degradation 137 kDa product (7.1%), and much higher non-IgG contaminants, notably albumin found in band 16 (Fig 2), accounting for 30.8% of the total antivenom proteins, and other plasma proteins in bands 8 (α_2_-macroglobulin, 7.9%), 12 and 13 (haptoglobin, 3.7%), 14 (serotransferrin, 4.1%), and 15 (α_1_B-glycoprotein, 3.7%) (Fig 2 and S1 Table).

Costa Rican ICP and Peruvian INS-PERU antivenoms displayed a very similar SDS-PAGE banding patterns and MS/MS-derived protein profiles, characterized by 80% intact IgGs, 3–4% IgG aggregates, 12–15% IgG degradation products, and minor non-IgG contaminants (0.9% vs 1.5% α_1_B-glycoprotein in bands 22 and 29, respectively; and 2.5% haptoglobin in band 27 of ICP antivenom) (Fig 2).

The F(ab')_2_ antivenoms manufactured in Venezuela (UCV) and Argentina (BIOL) exhibit similar qualitative SDS-PAGE profiles (Fig 2), though the latter represents a better purified product (88% F(ab')_2_, 7.3% IgG degradation product, and 4.4% non-IgG (fibrinogen γ-chain) contaminant) than the fabotherapic produced by the Universidad Central de Venezuela (70% F(ab')_2_, 9.9% non- processed IgG, 0.5% IgG aggregates, 13.3% IgG degradation products, and 6% α_2_-macroglobulin) (S1 Table).

The presence of IgG aggregates and non-IgG protein impurities contribute to the reduced safety profile of the products by increasing the possibility of early (anaphylactoid) reactions and anaphylactic shock due to IgE antibodies against these heterologous animal proteins [62,66–69]. The outcome of our present study indicates a need for improving the fractionation of the hyperimmune plasma, particularly the PROBIOL and UCV products.

### Immunoreactivity profile of antivenoms: third-generation antivenomics

**Fig 3:**
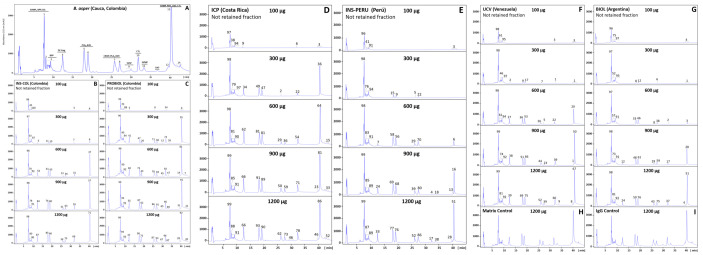
Immunocapture capacity of polyvalent antivenoms towards *B*. *asper* (*sensu stricto*) venom from Cauca, Colombia. Panel **A** displays the fractionation by reverse-phase HPLC of the venom components. Proteins eluting in each peak (1–14) were assigned using the venomics information reported by Mora-Obando *et al*. [32]. SVMP, snake venom Zn^2+^-metalloproteinase and their proteolytic fragments Disintegrin/Cysteine fragments (DC-frag); PLA_2_-K49/D49, phospholipase types Lys49 and Asp49; SVSP, serine proteinase; CTL, C-type lectin-like; DIS, disintegrin; CRISP, cysteine-rich secretory protein; LAO, L-amino acid oxidase; PDE, phosphodiesterase; SVMPi, SVMP inhibitors; BPP, bradykinin-potentiating-like peptides. Panels **B**-**G** represent RP-HPLC fractionations of the non-immunoretained fractions recovered in the flow-through fraction of the affinity columns of immobilized antivenoms INS-COL (**B**), PROBIOL (**C**), ICP (**D**), INS-PERU (**E**), UCV (**F**), BIOL (**G**) incubated with increasing amounts of venom (100–1200 μg). Panels **H** and **I** display to chromatographic separations of the venom fraction not retained in the mock matrix control and the naïve equine immunoglobulins control, respectively. The numbers on top of the chromatographic peaks represent the percentage of each fraction.

**Fig 4:**
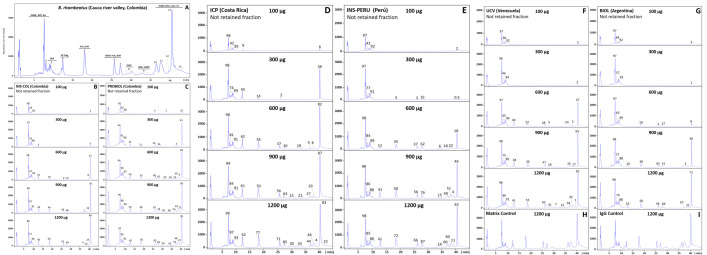
Immunocapture capacity of polyvalent antivenoms towards *B*. *rhombeatus* venom from Cauca river valley, Colombia. Panel **A** displays the fractionation by reverse-phase HPLC of the venom components. Proteins eluting in each peak (1–14) were assigned using the venomics information reported by Mora-Obando *et al*. [32]. Panels **B**-**G** represent RP-HPLC fractionations of the non-immunoretained fractions recovered from the affinity columns of immobilized antivenoms INS-COL (**B**), PROBIOL (**C**), ICP (**D**), INS-PERU (**E**), UCV (**F**), BIOL (**G**) incubated with increasing amounts of venom (100–1200 μg). Panels **H** and **I** display to chromatographic separations of the venom fraction not retained in the mock matrix control and the naïve equine immunoglobulins control, respectively. The numbers on top of the chromatographic peaks represent the percentage of each fraction.

**Fig 5:**
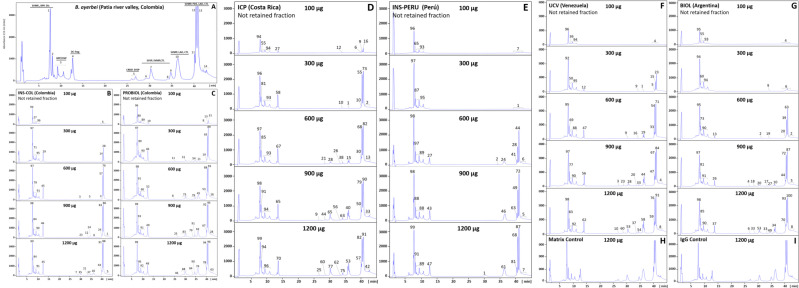
Immunocapture capacity of polyvalent antivenoms towards *B*. *ayerbei* venom from Patia river valley, Colombia (**A**). Panel **A** displays the fractionation by reverse-phase HPLC of the venom components. Proteins eluting in each peak (1–14) were assigned using the venomics information reported by Mora-Obando *et al*. [28]. Panels **B**-**G** represent RP-HPLC fractionations of the non-immunoretained fractions recovered from the affinity columns of immobilized antivenoms INS-COL (**B**), PROBIOL (**C**), ICP (**D**), INS-PERU (**E**), UCV (**F**), BIOL (**G**) incubated with increasing amounts of venom (100–1200 μg). Panels **H** and **I** display to chromatographic separations of the venom fraction not retained in the mock matrix control and the naïve equine immunoglobulins control, respectively. The numbers on top of the chromatographic peaks represent the percentage of each fraction.

The immunoreactivity of the six antivenoms against the venom components of *B*. *asper* (*sensu stricto*), *B*. *ayerbei*, and *B*. *rhombeatus*, previously identified by Mora-Obando *et al*. [28,32] was investigated by third-generation antivenomics [43–45] (Figs 3–5 and S1–S3). Table 3 summarizes the antivenoms' immunorecognition capabilities. All the antivenoms recognized around 12–14% of the low-molecular mass components eluted during the first 10 min in chromatographic fractions 1–3. These fractions comprise endogenous tripeptide inhibitors of snake venom metalloproteinases (SVMPi) [70–73], 10–12 amino acid residue bradykinin-potentiating-like peptides (BPPs) [72,74,75], and disintegrin (DIS) molecules. Among these early-eluting venom components only disintegrin are immunogenic [76–79], and therefore, the immunoretained fraction may comprise this class of toxins, which in *B*. *ayerbei* [28], *B*. *asper*, and *B*. *rhombeatus* [32] represent 2.3–5.6% of their total venom proteins. On the other hand, chromatographic fractions 4–7, eluted between 10–30 min, comprised disintegrin-like/cysteine-rich (DC) fragments of PIII-SVMPs, phospholipase A_2_ (PLA_2_) molecules, cysteine-rich secretory proteins (CRISP) and serine proteinases (SVSP), and were immunocaptured at maximal immunoaffinity column binding capacity with average efficacy of 62% (*B*. *asper*), 70% (*B*. *rhombeatus*), and 80% (*B*. *ayerbei*) (Table 3). Chromatographic fractions 8–14 eluted between 30–45 min contained SVMP, C-type lectin-like proteins (CTL), SVSP, L-amino acid oxidase (LAO), and phosphodiesterase (PDE), and were immunoretained in the affinity matrices with average efficacy of 80% (*B*. *asper*), 87% (*B*. *rhombeatus*), and 69% (*B*. *ayerbei*) (Table 3). Table 4 shows a summary of the maximum total toxin binding capacity of each of the *B*. *asper* linage venoms by the different antivenoms. Extrapolated to mg venom immunocaptured per gram of antivenom the ranking of antivenoms according to their respective highest to lowest binding capacity towards total components was [BIOL > INS-PERU > UCV > INS-COL > ICP > PROBIOL] for *B*. *asper* (*sensu stricto*), [BIOL > INS-COL > (UCV ~ INS-PERU) > ICP > PROBIOL] for *B*. *rhombeatus*, and [INS-COL > (BIOL ~ INS-PERU) > UCV > ICP > PROBIOL] for *B*. *ayerbei*. In terms of percentage of toxin-binding antibodies, the order from highest to lowest relative abundance was [INS-PERU > (BIOL ~ INS-COL) > UCV > ICP > PROBIOL] for *B*. *asper* (*sensu stricto*), [INS-COL > (BIOL ~ INS-PERU) > (ICP ~ UCV ~ PROBIOL)] for *B*. *rhombeatus*, and [(INS-COL ~ INS-PERU) > BIOL > ( ICP ~ UCV ~ PROBIOL)] for *B*. *ayerbei*.

**Table 3:** Percentages of NOT immunoretained fractions of *B*. *asper* venoms by six Latin American antivenoms.

Venom	Fractions*	Major venom components	% not immunoretained						
			INS-COL	PROBIOL	ICP	BIOL	UCV	INS-PERU	AVERAGE
*B*. *asper* (*sensu stricto*)	1–3	SVMPi, BPP, DIS	86	92	88	86	85	88	88
	4–7	DC-frag, PLA_2_, CRISP	40	51	50	23	30	31	38
	8–14	SVSP, SVMP, CTL, LAO, PDE	15	45	28	9	10	13	20
*B*. *rhombeatus*	1–3	SVMPi, BPP, DIS	84	88	88	85	84	87	86
	4–7	DC-frag, PLA_2_, CRISP	23	44	40	18	21	33	30
	8–14	SVSP, SVMP, CTL, LAO, PDE	8	28	17	5	11	13	13
*B*. *ayerbei*	1–3	SVMPi, BPP, DIS	87	93	91	86	83	91	88
	4–7	DC-frag, CRISP, SVSP	12	43	31	11	18	6	20
	8–14	SVSP, SVMP, CTL, LAO, PDE	21	54	38	25	27	24	31

The immunocapture efficiency of each antivenom is color-coded based on the percentage of the NOT immunoretained toxin fractions. Dark green: <25%, light green: 25–55%, light brown: 55–80% and red:> 80%. Polyvalent antivenoms manufactured by Instituto Nacional de Salud, Colombia (INS-COL); Laboratorios Probiol S.A., Colombia (PROBIOL); Instituto Clodomiro Picado, Costa Rica (ICP); Instituto Biológico Argentino S.A.I.C (BIOL); Centro de Biotecnología de la Unidad de Farmacia de la Universidad Central de Venezuela (UCV) and Instituto Nacional de Salud, Peru (INS-PERU). * Fraction numbers and abbreviations of the main venom components are described in Fig 3.

**Table 4:** Summary of third generation antivenomics analyses of polyvalent antivenoms against venoms of the three *B*. *asper* lineages from south-western Colombia.

Antivenom (antibody type)	mg/vial	Venom	Maximal binding capacity		ʃMolMass	Anti-toxin antibodies (%)		Lethality neutralizing antibodies	
			(mg V/g AV)	(mg V/ vial)		2 Ag-binding sites	1 Ag-binding site	% toxin-neutralizing Abs**	% (toxin-binding & neutralizing) Abs
INS-COL (IgG)	568	BAS	47.9	27.2	32.0	12.0	24.0	13.2	54.9
		BRH	66.2	37.6	28.0	18.9	37.8	16.9	44.6
		BAY	59.2	33.6	40.1	11.8	23.6	12.0	50.7
PROBIOL (IgG)	2007.4	BAS	20.0	40.1	33.8	4.7	9.5	2.5	26.7
		BRH	33.1	66.5	25.1	10.6	21.1	3.8	17.8
		BAY	25.3	50.8	36.0	5.6	11.2	4.1	36.0
ICP (IgG)	596.7	BAS	32.4	19.4	28.4	9.1	18.2	10.2	56.2
		BRH	41.2	24.6	27.9	11.8	23.6	12.0	50.7
		BAY	33.8	20.2	43.4	6.2	12.5	9.3	74.3
INS-PERU (IgG)	591.2	BAS	65.2	38.5	31.8	16.4	32.8	N.D	N.D
		BRH	53.5	31.6	30.8	13.9	27.8	N.D	N.D
		BAY	54.2	32.0	38.9	11.2	22.3	N.D	N.D
BIOL F(ab')_2_	592.6	BAS	71.3	42.3	31.4	12.5	25.1	N.D	N.D
		BRH	75.4	44.7	29.4	14.1	28.2	N.D	N.D
		BAY	54.4	32.3	42.0	7.1	14.3	N.D	N.D
UCV F(ab')_2_	419*	BAS	60.8	25.5	29.8	11.2	22.3	N.D	N.D
		BRH	57.8	24.2	28.1	11.3	22.6	N.D	N.D
		BAY	42.8	18.0	41.9	5.6	11.3	N.D	N.D

BAS: *B*. *asper* (*sensu stricto*), BRH, *B*. *rhombeatus;* BAY, *B*. *ayerbei;* V, venom; AV, antivenom; Ag, antigen; Abs, antibodies

* Dr. Mariana Cepeda (personal communication, 2020).

** % Lethality neutralizing Abs per vial/ % of total toxin-binding Abs per vial

**Fig 6:**
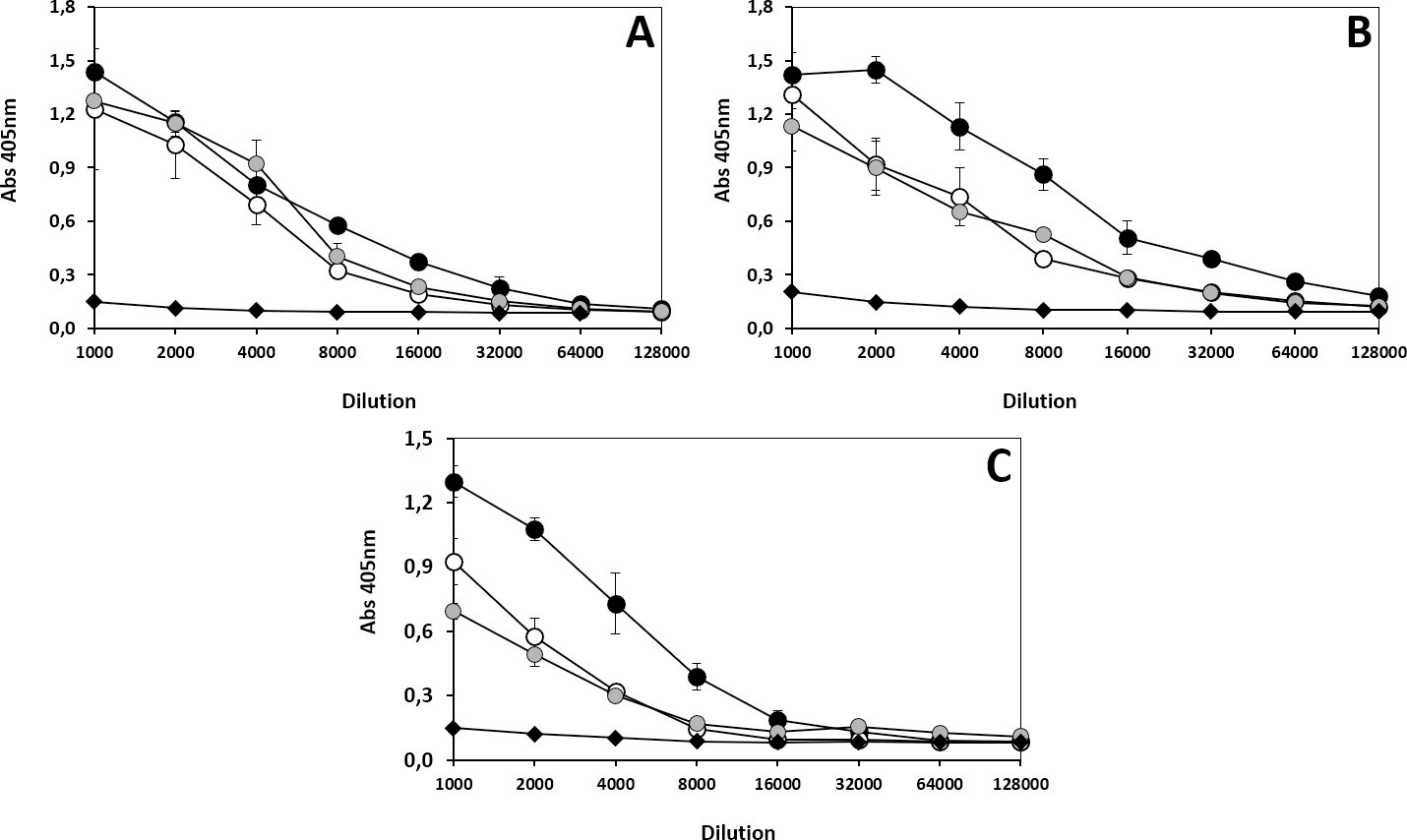
Titration curves of polyvalent antivenoms against the venoms of three lineages of *B*. *asper* venoms. Antivenoms INS-COL (●), PROBIOL (●), ICP (○) were serially diluted by a factor of two (starting from a dilution of 1/1000) and tested by ELISA against the following crude venoms: *B*. *asper* (*sensu stricto*) (**A**), *B*. *rhombeatus* (**B**) and *B*. *ayerbei* (**C**). Equine normal serum was included as negative control (◆). Each point represents the mean ± SD of three independent determinations. Statistically significant differences were observed among the titers of the antivenoms INS-COL vs. PROBIOL and/or ICP against the venoms of *B*. *asper* (dilutions 1: 1000, 8000, 16000, 64000), *B*. *rhombeatus* (dilutions 1:2000–128000) and *B*. *ayerbei* (dilutions 1:1000–16000).

Although none of the antivenom affinity matrices outshined all the others in its ability to immunocapture each and every one of the components of the three *B*. *asper* lineage venoms, the results displayed in S2–S19 Tables and summarized in Tables 3 and 4 clearly classify the antivenoms into two groups according to their antivenomics immunocapturing features, with [BIOL, INS-PERU, INS-COL and UCV] (61.3 ± 9.9 (BAS), 63.2 ± 9.7 (BRH), and 52.7 ± 6.9 (BAY) mgV/gAV) in the top group and [ICP > PROBIOL] (26.2 ± 8.8 (BAS), 37.2 ± 5.7 (BRH), and 29.5 ± 6.0 (BAY) mgV/gAV) in the least effective group. The same trend was observed when the immunoreactivity of INS-COL, ICP, and PROBIOL against the three *B*. *asper* lineage venoms was comparatively assessed by ELISA: INS-COL showed the highest titer against all the venoms and the lower levels of cross-reactivity of ICP and PROBIOL against the three venoms were similar and indistinguishable between themselves (Fig 6). In addition, comparison of the relative amounts (%) of anti-toxin antibodies in the different antivenoms (Table 4), calculated assuming one antigen binding site occupied per immobilized antibody molecule, revealed that, on average, 28.5 ± 8.1%, 27.6 ± 5.2%, and 22.5 ± 7.3% of INS-COL, INS-PERU, and BIOL antivenom antibodies, respectively, recognized antigenic determinants on toxins from each of the three *B*. *asper* lineage venoms, whereas these figures were 18.7 ± 6.5%, 18.1 ± 5.6% and 13.9 ± 6.3% in the case of UCV, ICP and PROBIOL antivenoms, respectively. INS-COL, PROBIOL and ICP showed higher percentages of anti-BRH than anti-BAS and anti-BAY antibodies, whereas BIOL and UCV contained equivalent relative amounts of anti-BAS and anti-BRH, but significantly lower % of anti-BAY antibodies, and the relative amounts of anti-toxin antibodies in INS-PERU were BAS > BRH > BAY (Table 4). These figures fall within the range of percentages (6–28%) of anti-toxin antibodies determined for other commercial antivenoms [48,51,52,79–81]. The distinct cross-reactive profiles of the different bothropic antivenoms may be ascribed to the immunization process, in particular to the use of venoms from different *Bothrops* species or from different geographic variations of the same nominal species used by the different manufacturers, i.e. INS-COL and PROBIOL (*B*. *atrox*, *B*. *asper*), INS-PERU (*B*. *atrox*, *B*. *pictus*, *B*. *barnetti*, *B*. *brazili* [82]), ICP (Costa Rican *B*. *asper* from Caribbean and Pacific populations), UCV (*B*. *atrox*, *B*. *colombiensis*, *B*. *venezuelensis*), BIOL (*B*. *asper* of undisclosed geographic origin) [63].

### Neutralization of the biological effects of *B. asper* venoms by INS-COL, PROBIOL, and ICP antivenoms

#### Lethality

**Fig 7:**
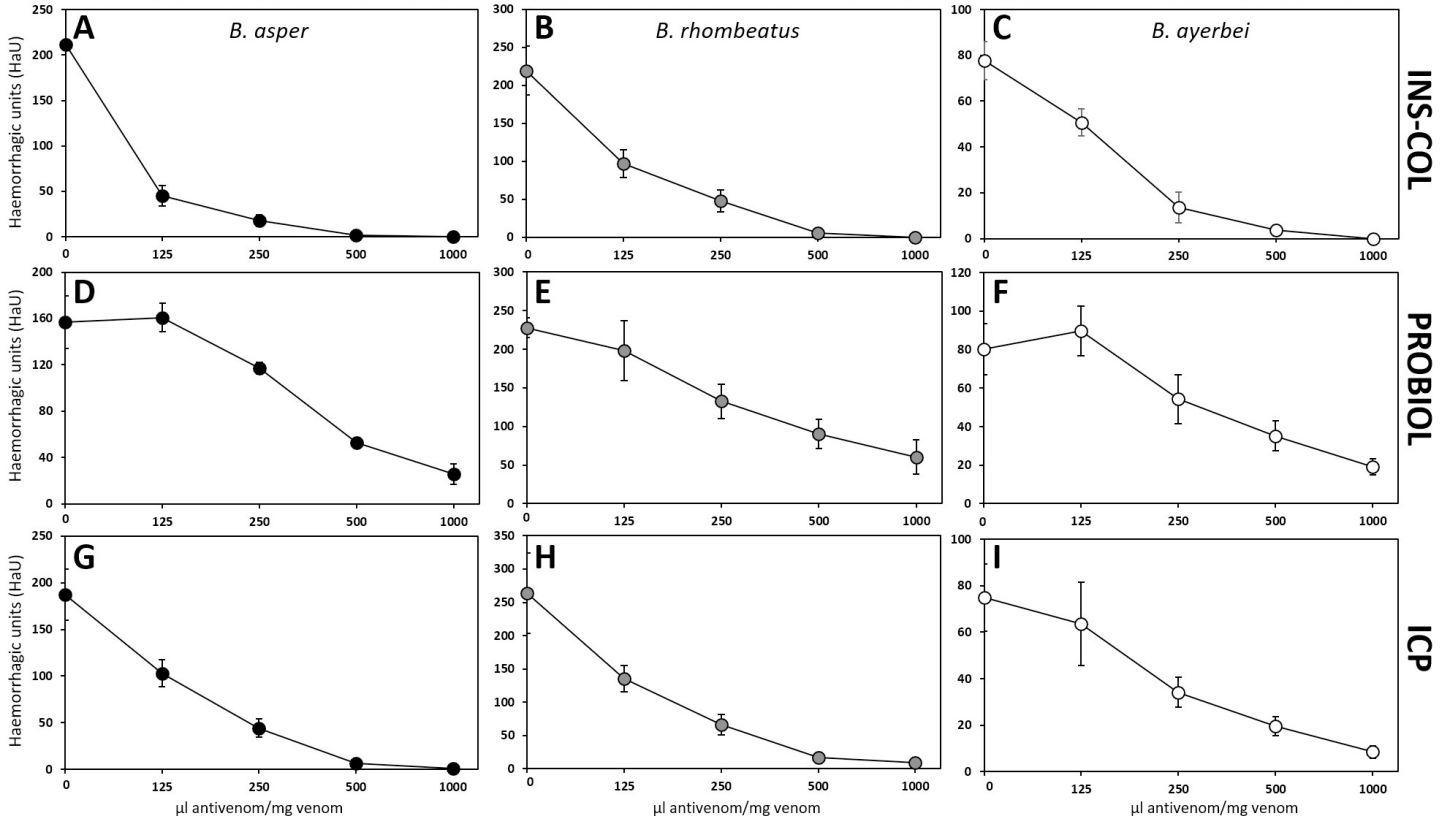
Comparison of the neutralizing capacity of the antivenoms manufactured by INS-COL (A-C), PROBIOL (D-F), ICP (G-I) towards the hemorrhagic effect caused by the venoms of *B*. *asper* (*sensu stricto*) (●), *B*. *rhombeatus* (●), *B*. *ayerbei* (○). Hemorrhagic lesions were measured as hemorrhagic units (HaU) [55] 2 h after intradermal injection of 10 MHD (challenge dose) mixed with antivenom in the ratios indicated in the figure. Each point represents the mean ± SD of four replicates. In all assays, statistically significant differences (p<0.05) were observed among the ratios 500–1000 μL antivenom/mg venom compared to the positive control.

**Fig 8:**
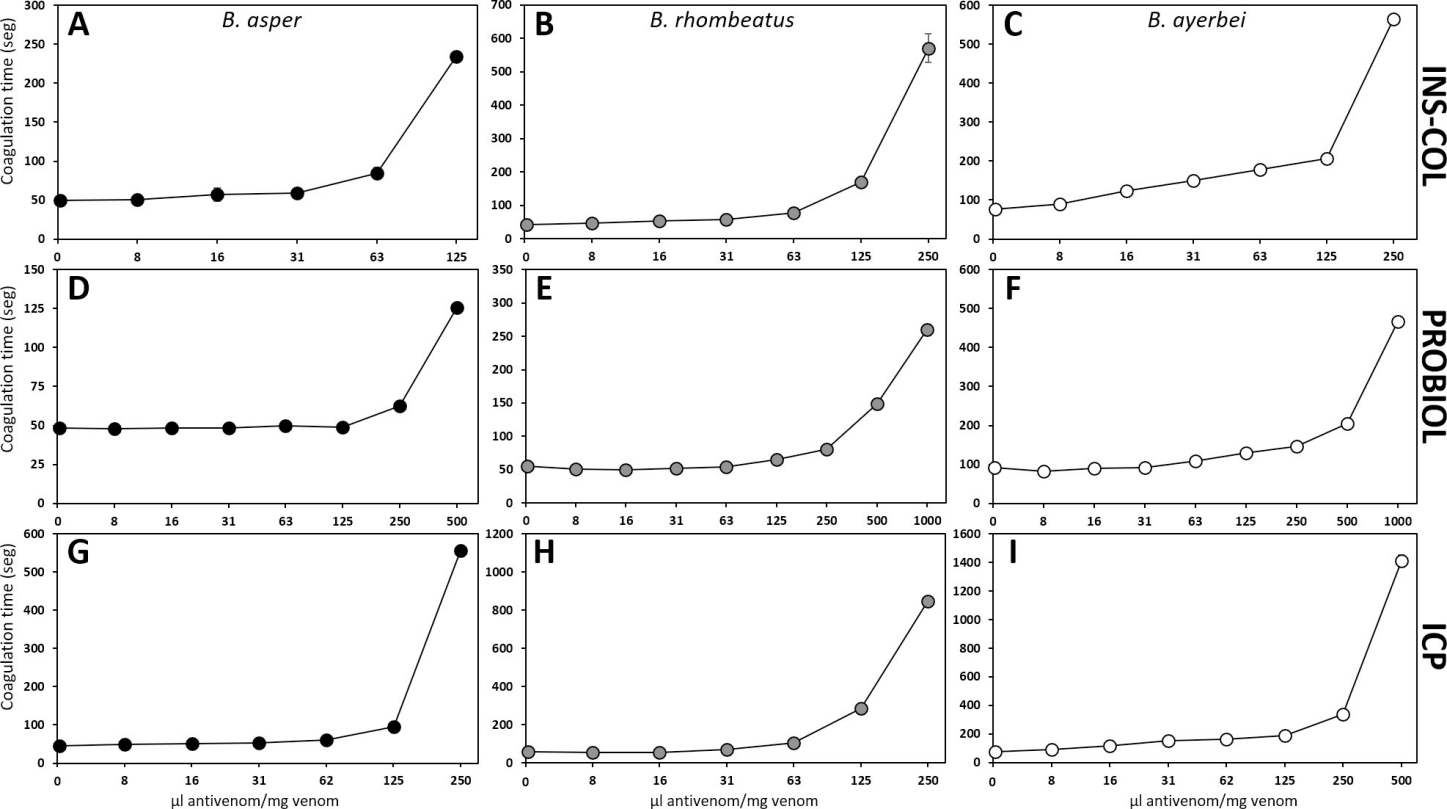
Comparison of the neutralizing capacity of the antivenoms manufactured by INS-COL (A-C), PROBIOL (D-F), ICP (G-I) towards the coagulant effect caused by the venoms of *B*. *asper* (*sensu stricto*) (●), *B*. *rhombeatus* (●), *B*. *ayerbei* (○). Clotting time was recorded after mixing and incubating 2 MCD (challenge dose) with different ratios of antivenom, as indicated in the figure, and adding them to human citrated plasma. Each point represents the mean ± SD of replicates of two independent experiments.

**Fig 9:**
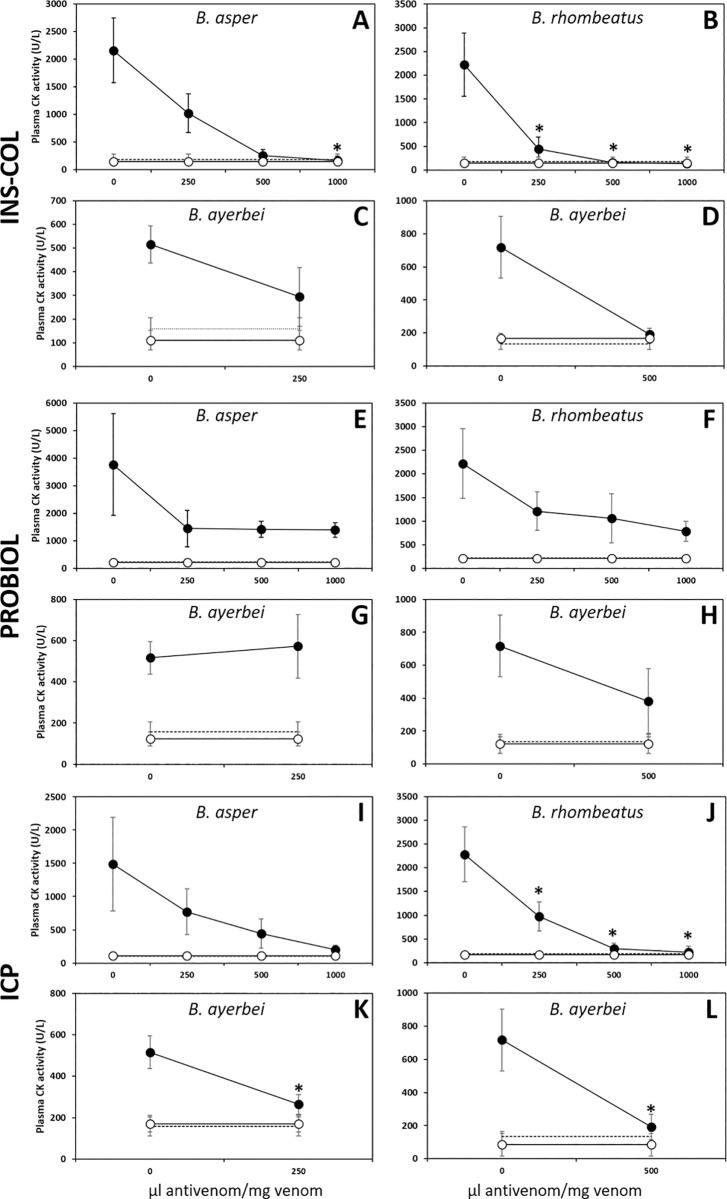
Comparison of the neutralizing capacity of the antivenoms manufactured by INS-COL (A-D), PROBIOL (E-H), ICP (I-L) towards the myotoxic effect caused by the venoms of *B*. *asper* (*sensu stricto*) (A, E, I), *B*. *rhombeatus* (B, F, J), *B*. *ayerbei* (C, D, G, H, K, L). Muscle damage was measured as plasma creatine kinase activity 3 h after intramuscular injection of 50 μg of venom mixed with antivenom in the ratios indicated in the figure (●). Antivenom control (○). PBS control (---). Each point represents the mean ± SD of four replicates. Statistically significant differences (p<0.05) compared to the venom control are represented by asterisks.

**Fig 10:**
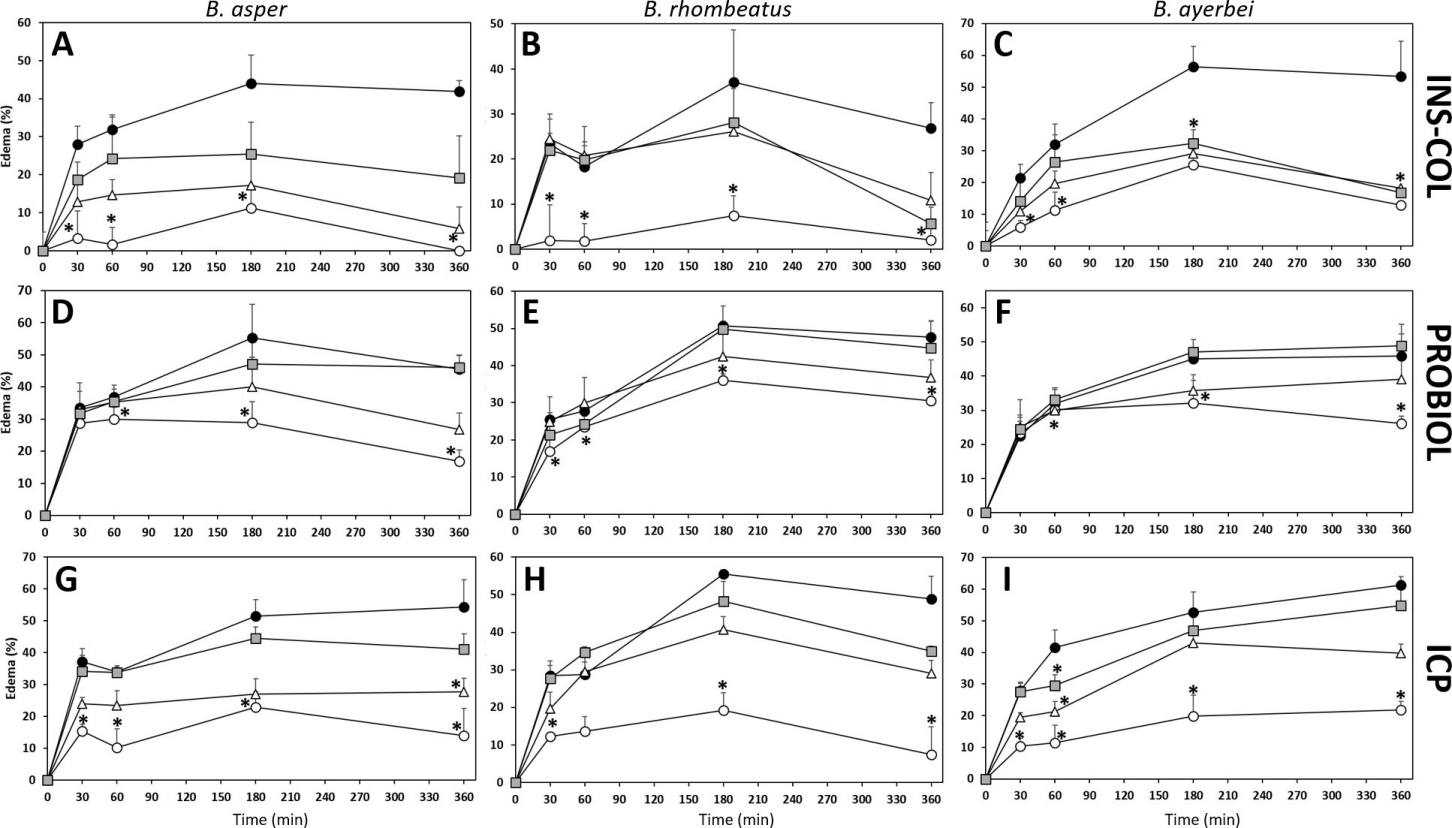
Comparison of the neutralizing capacity of the antivenom manufactured by INS-COL (A-C), PROBIOL (D-F), ICP (G-I) towards the edematogenic effect caused by the venom of *B*. *asper* (*sensu stricto*) (A, D, G), *B*. *rhombeatus* (B, E, H) and *B*. *ayerbei* (C, F, I). Edema was measured as the increase in the thickness of footpad compared to the negative control (PBS) and monitored during 6 h after subcutaneous injection of 5 μg of venom (●) mixed with antivenom in the ratios: 1000 μL antivenom/mg venom (○), 500 μL antivenom/mg venom (Δ), 250 μL antivenom/ mg venom (■). Each point represents the mean ± SD of four replicates. Statistically significant differences (p<0.05) compared to the venom control are represented by asterisks.

**Fig 11:**
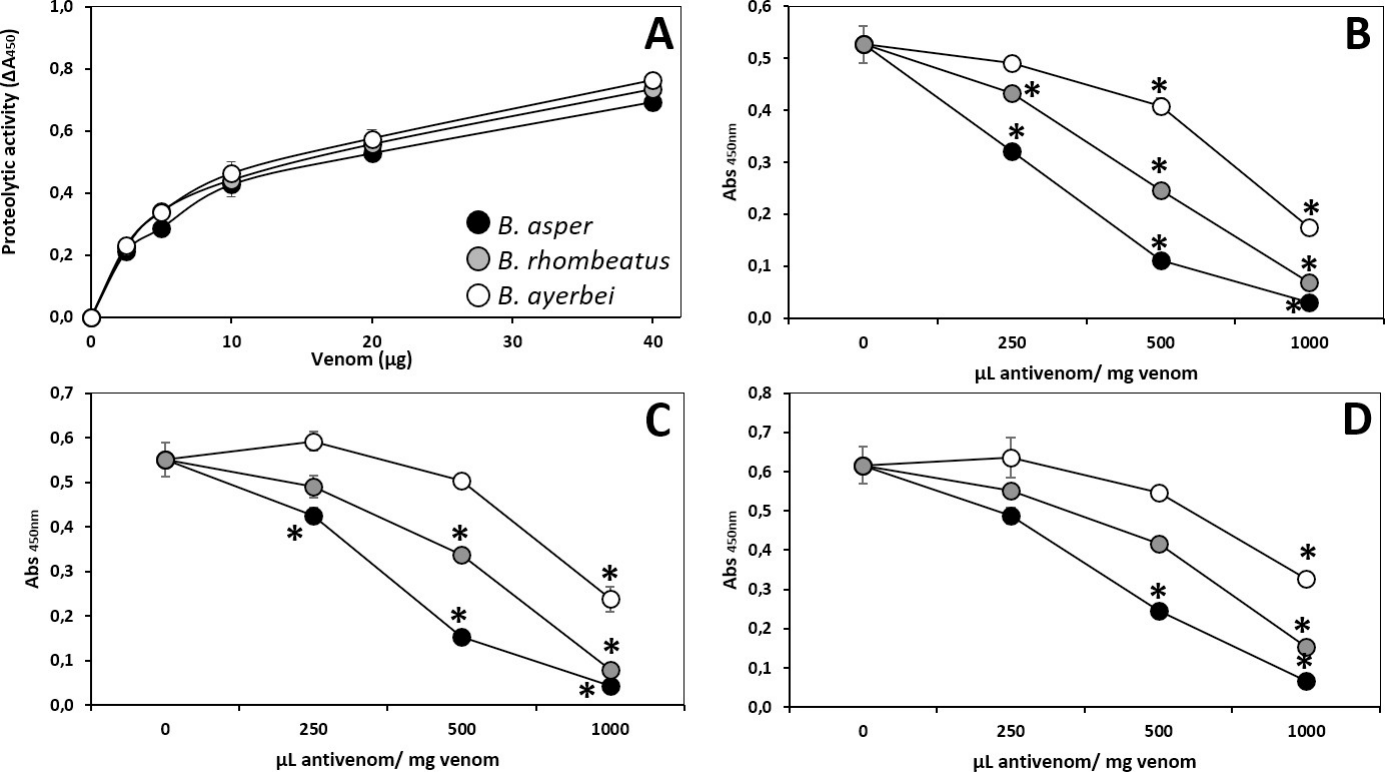
Proteolytic activity of the venoms of *B*. *asper* (*sensu stricto*), *B*. *rhombeatus*, and *B*. *ayerbei* (A) and neutralizing capacity of the antivenoms manufactured by INS-COL (●), PROBIOL (○) and ICP (●) towards *B*. *asper* (B), *B*. *rhombeatus* (C) and *B*. *ayerbei* (D). Neutralization of the proteolytic activity was measured on azocasein, as described in the Material and methods section, 90 min after mixing and incubating 12.5 μg of venom (challenge dose) with antivenom in the ratios indicated in the figure. Each point represents the mean ± SD of three replicates. Statistically significant differences (p<0.05) compared to the venom controls are represented by asterisks.

**Fig 12:**
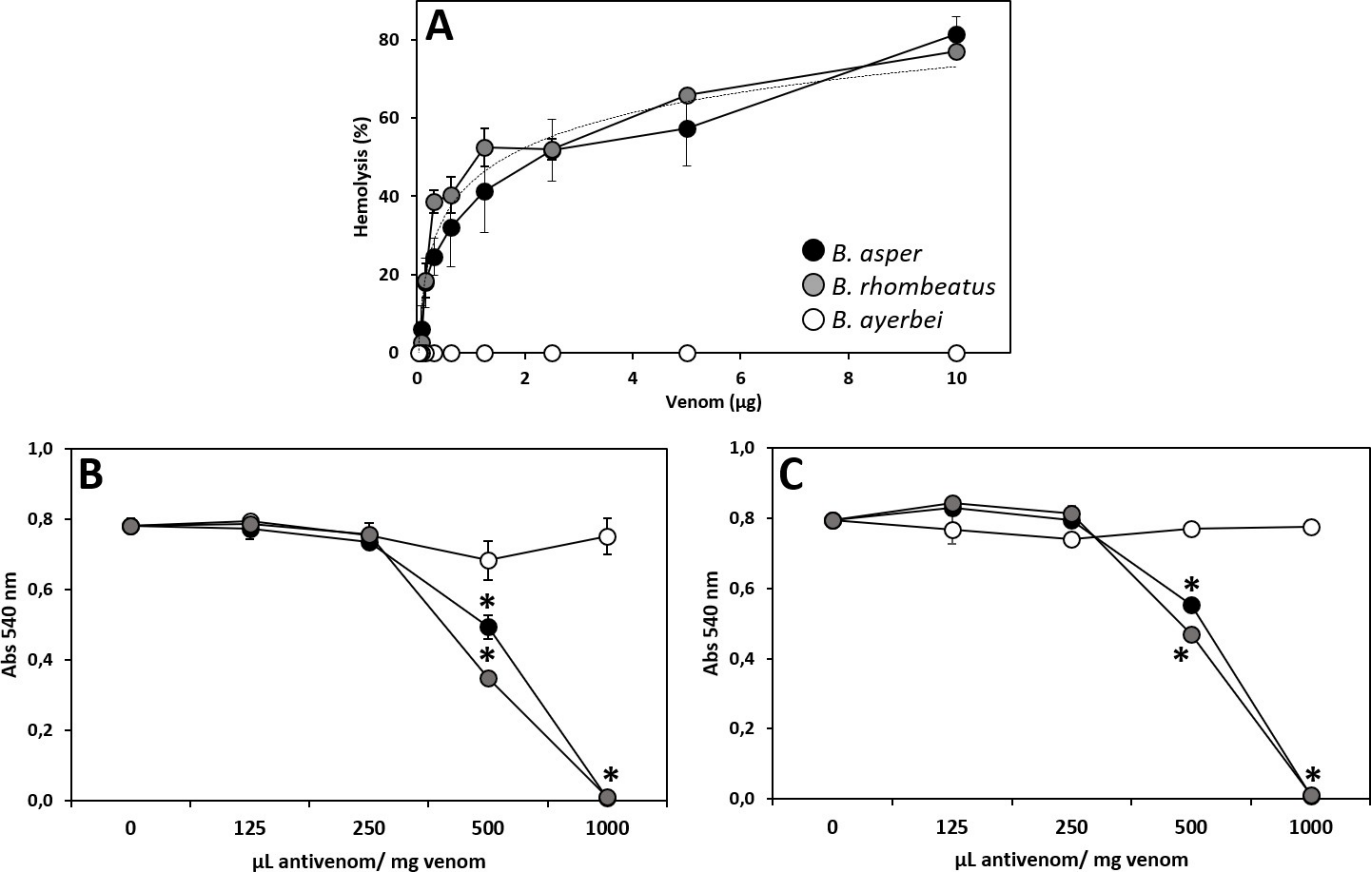
Indirect hemolytic effect produced by venoms of *B*. *asper*, *B*. *rhombeatus*, and *B*. *ayerbei* (A) and neutralizing capacity of the antivenoms INS-COL (●), PROBIOL (○) and ICP (●) towards *B*. *asper* (*sensu stricto*) venom (B) and *B*. *rhombeatus* (C). Neutralization of indirect hemolysis was measured on rabbit erythrocytes, as described in the Material and methods section, 1 h after mixing and incubating of 5.1 μg of venom (challenge dose) with antivenom in the ratios indicated in the figure. Each point represents the mean ± SD of three replicates. Statistically significant differences (p<0.05) compared to the venom control are represented by asterisks.

As a rule of thumb, immunocapturing capacity ≥ 20–25% of total venom proteins has been proposed to correlate with a promising outcome in an *in vivo* lethality neutralization assay [83], and increasing percentages of immunocapturing indicate greater neutralizing potencies of the corresponding bait antivenoms. Table 5 summarizes the results of the neutralizing capacities of the Colombian antivenoms INS-COL and PROBIOL and the Costa Rican ICP towards the lethal, hemorrhagic (Fig 7), coagulant (Fig 8), defibrinogenating, myotoxic (Fig 9), edematogenic (Fig 10), proteolytic (Fig 11), and indirect hemolytic (Fig 12) effects of the venoms of SW Colombian *B*. *asper* (*sensu stricto*), *B*. *rhombeatus*, and *B*. *ayerbei*. All three antivenoms followed the "antivenomics rule" regarding their potency to neutralize the lethal effect of the three *B*. *asper* lineage venoms, as well as the coagulant and proteolytic effects (Table 5). Antivenoms INS-COL and ICP showed statistically indistinguishable capacities to neutralize the indirect hemolytic effect of *B*. *asper* and *B*. *rhombeatus* venoms, the coagulant effect of *B*. *rhombeatus* venom, and the defibrinogenating and myotoxic effects of *B*. *rhombeatus* and *B*. *ayerbei* venoms (Table 5).

**Table 5:** Neutralization of biological activities of *B*. *asper* venoms by polyvalent antivenoms INS-COL, PROBIOL and ICP.

Venom	Activity	Challenge dose^1^ [μgV]	ED_50_/ED^2^ of activities neutralized by polyvalent antivenoms					
			INS-COL		PROBIOL		ICP	
			[mg V/mL AV]	[mg V/g AV]	[mg V/mL AV]	[mg V/g AV]	[mg V/mL AV]	[mg V/g AV]
*B*. *asper sensu stricto*	Lethality	403.6	5.0 (3.4–7.5)	71.4 (48.6–107.1)	1.0 (0.6–1.6)	14.3 (8.6–22.9)	3.4 (2.6–4.3)	48.6 (37.1–61.4)
	Potency		3.75	53.6	0.75	10.7	2.55	36.4
	Hemorrhage	14.4	13.4 ± 1.2^a,b^	191.4 ± 17.1	3.2 ± 0.30^c^	45.7 ± 4.3	6.7 ± 0.64	95.7 ± 9.1
	Coagulation	0.74	10.4 ± 0.06^a^	148.6 ± 0.9	1.6 ± 0.001^c^	22.9 ± 0.01	7.8 ± 0.05	111.4 ± 0.7
	Defibrinogenation	4	4.0	57.1	1.0	14.3	2.0	28.6
	Myotoxicity	50	5.5 ± 1.5^a^	78.6 ± 21.4	2.1 ± 0.2^c^	30 ± 2.9	3.3 ± 0.6	47.1 ± 8.6
	Edematogenic	5	2.15 ± 0.28	30.7 ± 4.0	N.N	N.N	1.30 ± 0.25	18.6 ± 3.6
	Proteolytic	12.5	3.48 ± 0.07^a^	49.7 ± 1	1.26 ± 0.1	18 ± 1.4	2.05 ± 0.4	29.3 ± 5.7
	Hemolytic	5.1	1.68 ± 0.09	24 ± 1.3	N.N	N.N	1.72 ± 0.03	24.6 ± 0.4
***B*. *rhombeatus***	Lethality	219.6	5.6 (5.0–6.3)	80 (71.4–90)	1.1 (0.6–2.0)	15.7 (8.6–28.6)	3.9 (2.6–5.8)	55.7 (37.1–82.9)
	Potency		4.20	60.00	0.83	11.8	2.93	41.8
	Hemorrhage	35.5	8.2 ± 0.85^a^	117.1 ± 12.1	3.1 ± 0.62^c^	44.3 ± 8.9	9.5 ± 0.64	135.7 ± 9.1
	Coagulation	0.42	9.7 ± 0.18^a^	138.6 ± 2.6	1.4 ± 0.001^c^	20 ± 0.01	9.3 ± 0.06	132.9 ± 0.9
	Defibrinogenation	6	2.0	28.6	1.0	14.3	2.0	28.6
	Myotoxicity	50	8.9 ± 1.6^a^	127.1 ± 22.9	2.1 ± 0.7^c^	30 ± 10	7.7± 3.7	110 ± 52.9
	Edematogenic	5	1.48 ± 0.14	21 ± 2.0	N.N	N.N	1.00 ± 0.18	14.3 ± 2.6
	Proteolytic	12.5	3.00 ± 0.04^a^	42.9 ± 0.6	0.88 ± 0.03	12.6 ± 0.4	1.55 ± 0.03	22.1 ± 0.4
	Hemolytic	5.1	1.65 ± 0.03	23.6 ± 0.4	N.N	N.N	1.66 ± 0.03	23.7 ± 0.4
***B*. *ayerbei***	Lethality	200.4	5.7 (4.9–6.6)	81.4 (70–94.3)	1.7 (0.7–2.4)	24.3 (10–34.3)	4.7 (4.4–5.0)	67.1 (62.9–71.4)
	Potency		4.28	61.1	1.28	18.2	3.53	50.4
	Hemorrhage	2.4	7.2 ± 0.68^a,b^	102.9 ± 9.7	2.7 ± 0.39	38.6 ± 5.6	4.6 ± 1.12	65.7 ± 16
	Coagulation	1.92	8.4 ± 0.06^a^	120.0 ± 0.9	1.5 ± 0.001^c^	21.4 ± 0.01	6.4 ± 0.01	91.4 ± 0.1
	Defibrinogenation	6	1.0	14.3	0.5	7.1	1.0	14.3
	Myotoxicity	50	4.4 ± 1.3^a^	62.9 ± 18.6	1.6 ± 0.9^c^	22.9 ± 12.9	4.8 ± 0.9	68.6 ± 12.9
	Edematogenic	5	1.49 ± 0.67	21.3 ± 9.6	N.N	N.N	1.98 ± 0.54	28.7 ± 7.7
	Proteolytic	12.5	2.4 ± 0.44^a^	34.3 ± 6.3	0.99 ± 0.06	14.1 ± 0.9	1.33 ± 0.02	19 ± 0.3
	Hemolytic	5.1	N.D	N.D	N.D	N.D	N.D	N.D

For comparative purposes, neutralization activities were carried out with previously dialyzed and lyophilized antivenoms for antivenomics experiments prepared at a stock concentration of 70 mg/mL. Polyvalent antivenoms manufactured by Instituto Nacional de Salud, Colombia (INS-COL); Laboratorios Probiol, Colombia (PROBIOL); Instituto Clodomiro Picado, Costa Rica (ICP). ^1^Table 2 describes the reference doses and the number of doses used to calculate the challenge dose. ^2^Neutralization of lethal, hemorrhagic, myotoxic, edematogenic, proteolytic and hemolytic activities is expressed as median effective dose (ED_50_) and neutralization of coagulant and defibrinogenating activities is expressed as Effective Dose (ED). Potency was calculated as [(n-1)/ ED_50_]×LD_50_ (see Materials and Methods section for details). Doses in μL antivenom/mg venom were converted to mg venom (V)/mL antivenom (AV) and mg V/g AV. The significant differences among groups, INS-COL vs. PROBIOL, INS-COL vs. ICP, PROBIOL vs. ICP are represented by letters a, b, and c (superscripts) respectively. N.D: non-determined. N.N: the effect was not neutralized to 50% even with the highest antivenom/venom ratio.

The fraction of toxin-binding antibody molecules present in the antivenoms INS-COL, PROBIOL, and ICP, which contributed to protect the test animals from the lethal effect of the three *B*. *asper* lineage venoms, was derived by dividing the percentage of neutralizing antibodies (calculated from the antivenom's potency, S20 Table) by the percentage of toxin-binding antibodies (calculated from the maximal total venom protein binding capacity of the antivenom) (*B*. *asper*, S2–S4 Tables; *B*. *rhombeatus*, S8–S10 Tables; *B*. *ayerbei*, S14–S16 Tables). For these calculations, it was assumed that antibodies immobilized in the affinity columns bound on average one antigen molecule per IgG/F(ab')_2_ molecule, while in solution the same antibodies would have their two antigen-binding sites occupied. These conditions are based on the most coherent fitting of the data from a number of combined antivenomics and *in vivo* neutralization studies carried out in our laboratory during the last years. The results listed in Table 4 unveiled that 60.4 ± 12.3%, 50.1 ± 5.2% and 26.8 ± 9.1% of the toxin-binding antibodies of ICP, INS-COL, and PROBIOL contribute to the lethality neutralization activity of these antivenoms, respectively. Expressed as relative abundance of toxin-neutralizing antibodies per vial, INS-COL contained the highest value (14.0 ± 2.6%), followed by ICP (10.5 ± 1.4%) and PROBIOL (3.5 ± 0.9%) (Table 4). The potency (in mg of venom neutralized per gram of antivenom antibodies) of INS-COL, ICP and PROBIOL were roughly similar for the three *B*. *asper* lineage venoms (Table 5), with average values of 58.2 ± 4.1, 42.9 ± 7.1, and 13.6 ± 4.1 mg V/g AV, respectively.

#### Venoms' toxic activities

Hemorrhage induced by the three venoms was almost completely neutralized by INS-COL and ICP antivenoms at a ratio of 1000 μL antivenom/mg venom (Fig 7A–7C and 7G–7I). At the same ratio, a hemorrhagic lesion between 50 and 100 mm^2^ was observed with PROBIOL antivenom (Fig 7D–7F). Except for the *B*. *rhombeatus* venom's lethal effect, which was neutralized with statistically indistinguishable ED_50_ by INS-COL and ICP, the neutralizing efficacy of the antivenoms followed the order INS-COL > ICP > PROBIOL (Table 5). The antivenoms followed the same trend regarding their coagulant, defibrinogenating neutralization activities (Table 5). In this sense, INS-COL and ICP antivenoms extended the coagulation time above 30 min at a ratio of 250 μL antivenom/mg venom (Fig 8A–8C and 8G–8I), while even at a ratio of 500 μL antivenom/mg venom PROBIOL did not triple the coagulation time compared to the positive control (Fig 8D–8F). The ED_50_ of PROBIOL to neutralize the coagulant and the defibrinogenating activity of the three venoms was 2–4 times less effective than those of INS-COL and ICP (p<0.05) (Table 5).

The myotoxic effect of the venoms was nearly abrogated by INS-COL and ICP antivenoms at a ratio of 1000 μL antivenom/mg venom (Fig 9A–9D and 9I–9L). Conversely, PROBIOL antivenom only showed an average neutralization capacity of about 60% at the highest antivenom/venom ratios tested (Fig 9E–9H). Consequently, the ED_50_s of INS-COL and ICP are statistically indistinguishable from each other and significantly (p<0.05) higher (more effective) than that of PROBIOL towards each Colombian *B*. *asper* lineage venom tested (Table 5).

Both INS-COL and ICP antivenoms effectively neutralized, albeit the Colombian product exhibiting slightly higher ED_50_ compared to the Costa Rican antivenom, the edematogenic effect of *B*. *asper* (*sensu stricto*), *B*. *rhombeatus*, and *B*. *ayerbei* venoms (Table 5). However, even at the highest antivenom/venom ratio tested, PROBIOL was unable to reduce the effect to 50% (Table 5). Edematogenic activity curves, in which the injected animals were monitored during 360 min (Fig 10A–10I), showed that both INS-COL and ICP antivenoms reduced, at the highest ratio of antivenom tested and within the first 30 min, > 50% of the edema produced by all the venoms (Fig 10A–10C and 10G–10I). On the contrary, PROBIOL was only capable of partly neutralizing (by 30%) the edema triggered by *B*. *rhombeatus* venom (Fig 10D–10F). In general, the neutralizing effect of antivenoms was maintained six hours after venom injection (Fig 10); besides, INS-COL and ICP antivenoms neutralized the vasculotoxic effect produced by the venom, contributing in parallel to the neutralization of bleeding.

Interestingly, in some experiments involving PROBIOL antivenom/venom mixtures the edema increased above the size of the positive control (mice injected with venom alone) (Fig 10D–10F). Previous studies performed with *B*. *asper* venom from Costa Rica have also described this circumstance [84]. Proteolytic release of vasoactive peptides from serum protein contaminants (i.e., α_2_-globulins) during the incubation time of the antivenom/venom mixture, affecting vascular permeability and edema formation, has been invoked to explain this phenomenon. The fact that this event was mainly observed in experiments involving the antivenom with the most impurities of plasma proteins, PROBIOL (Fig 1 and S1 Table), would support this explanation.

Proteolytic activity of the three venoms was similar (Fig 11A). This effect is attributed mainly to the action of serine proteinases and Zn^2+^-dependent SVMPs present in *B*. *asper* venom [50]. INS-COL antivenom effectively neutralized the proteolytic activity of the three Colombian *B*. *asper* lineage venoms (ED_50_ = 42.3 ± 7.7 mg venom/g antivenom), followed in order of potency by ICP and PROBIOL (23.5 ± 5.3, and 14.9 ± 2.8 mg venom/g antivenom), respectively (Fig 11B and 11C and Table 5).

Indirect hemolytic activity was recorded in *B*. *asper* (*sensu stricto*) and *B*. *rhombeatus* venoms, but not in *B*. *ayerbei* venom (Fig 12A), as has been previously noted [28]. This effect is associated to high abundance of myotoxic PLA_2_s [85] and was quantified measuring the venom's phospholipase activity on phosphatidylcholine using erythrocyte lysis as an indicator [86]. The equivalent indirect hemolytic activity of *B*. *asper* (*sensu stricto*) and *B*. *rhombeatus* (Fig 12B) venoms was neutralized by INS-COL and ICP antivenoms with similar ED_50_ of 24.0 ± 0.5 mg venom/g antivenom (Table 5), while PROBIOL did not show neutralization efficacy of the indirect hemolytic activity of *B*. *asper* (*sensu stricto*) and *B*. *rhombeatus* venoms even at a ratio of 2000 μL antivenom/mg venom.

## Concluding remarks

Our data confirm and expand previous studies. Otero *et al*. (2002) reported a comparative study of the neutralizing capacity of four polyvalent antivenoms manufactured in Colombia (INS-COL, PROBIOL), Venezuela (UCV), and México (Antivipmyn, Instituto Bioclon) against the pharmacological and enzymatic effects of the venoms of *B*. *asper* and *Porthidium nasutum* from Antioquia (northwest Colombia) and Chocó (west Colombia) [38]. In their study, INS-COL and Antivipmyn antivenoms showed the highest neutralizing efficacy against the lethal and other pharmacological (hemorrhagic, edema-forming, myonecrotic, defibrinogenating and indirect hemolytic) effects of *B*. *asper* venom pooled from 40–45 specimens from different regions of Antioquia and Chocó. Conversely, antivenom PROBIOL presented the lowest neutralizing capacity towards the same activities, and the Venezuelan UCV antivenom had intermediate neutralization abilities. Understanding the basis of the effectivity of antivenoms against homologous and heterologous venoms demands the quantitative assessment of its toxin-resolved immunorecognition profile. Here we have applied third-generation antivenomics to compare the specific and paraspecific immunoreactivity of six bothropic antivenoms against three Colombian *B*. *asper* lineage venoms. The antivenomics outcome showed that all the major toxin families, i.e., SVMP, PLA_2_, CRISP, SVSP, CTL [32], were immunocaptured at maximal immunoaffinity column binding capacity with average efficacy of 62–87% (Table 3). These results clearly indicate that the paraspecificity exhibited by the six bothropic antivenoms against the three Colombian *B*. *asper* lineage venoms is not biased towards any particular family of toxins but is well balanced among the different venom protein families. Therefore, our study provides a solid experimental ground to rationalize the reported immunological profiles of INS-COL, UCV, and PROBIOL [38], and other bothropic antivenoms (INS-PERU, ICP, and BIOL), against *B*. *asper* venoms from different geographic Colombian ecoregions. These results strongly suggest the feasibility of adding these antivenoms to the list of candidates for the treatment of snakebite accidents caused by the species of the *B*. *asper* complex from SW Colombia, *B*. *asper* (*sensu stricto*), *B*. *rhombeatus* and *B*. *ayerbei* in the Departments of Nariño and Cauca.

**Fig 13:**
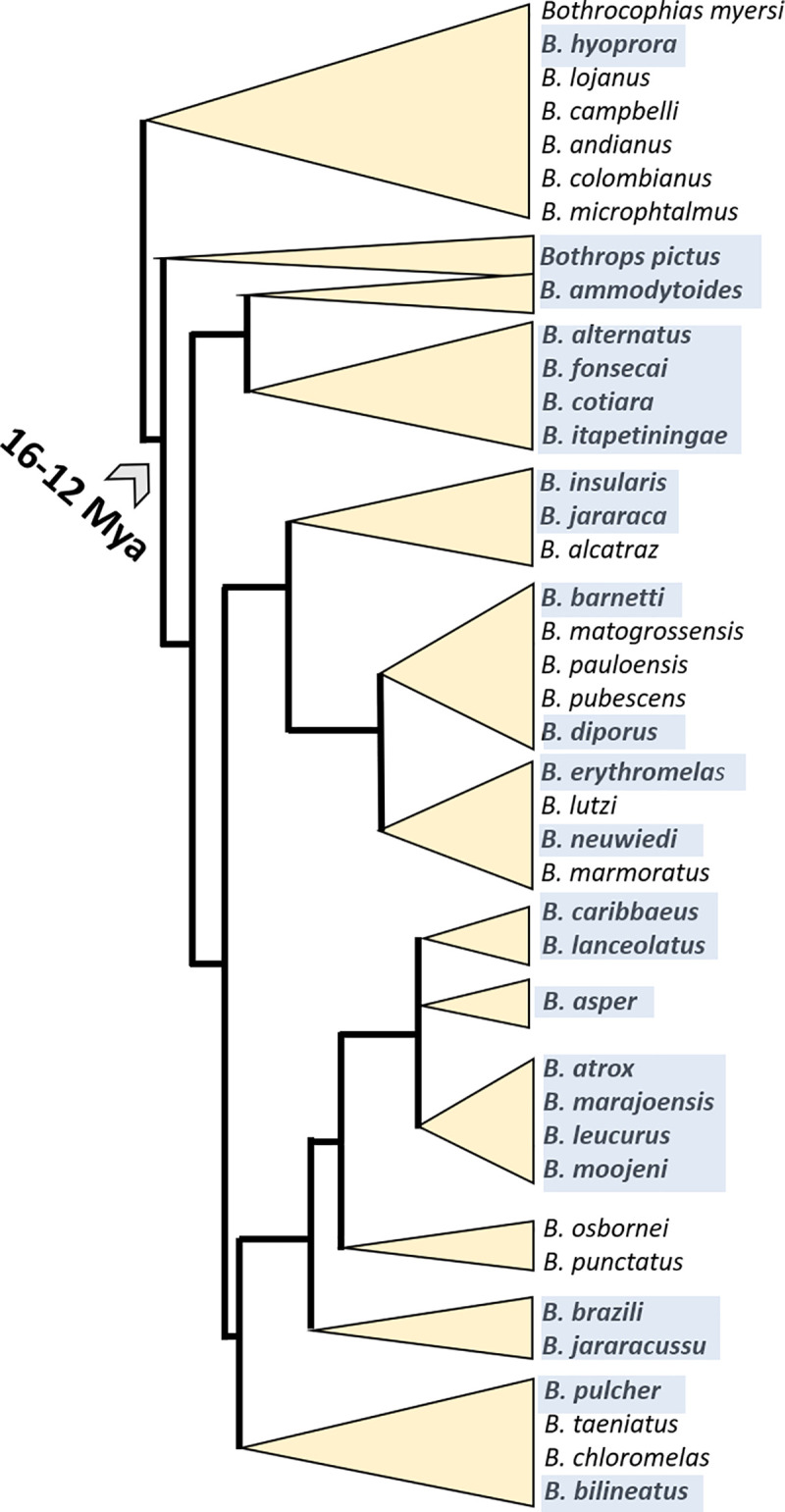
Phylogenetic tree of *Bothrops* highlighting some species reported by [88], whose venoms have been shown to exhibit remarkable immunoreactivity towards homologous and heterologous antivenoms produced in different Latin American countries using immunization mixtures that include different bothropic venoms.

Queiroz and co-workers [87] have reported *in vitro* qualitative (Western blot) and semi-quantitative (ELISA) evidence that Brazilian polyspecific pentabothropic (SAB) or antibothropic-lachesic F(ab')_2_ antivenoms exhibited variable paraspecific immunoreactivity towards nineteen venoms of bothropic snakes, including *B*. *brazili*, *B*. *alternatus*, *B*. *atrox*, *B*. *bilineatus*, *B*. *castelnaudi*, *B*. *cotiara*, *B*. *erythromelas*, *B*. *fonsecai*, *B*. *insularis*, *B*. *itapetiningae*, *B*. *jararaca*, *B*. *jararacussu*, *B*. *leucurus*, *B*. *marajoensis*, *B*. *moojeni*, *B*. *neuwiedi*, *B*. *pirajai*, *B*. *pradoi*, and *Bothrocophias hyoprorus*. The remarkable para-specificity exhibited by antivenoms generated against immunization mixtures that included venoms from phylogenetic distant *Bothrops* species may be ascribed to large conservation of immunoreactive epitope on venom toxins across much of the natural history of *Bothrops*, a genus that had its roots in South America during the middle Miocene, 14.07 (CI_95_: 16.37–11.75) Mya [88,89] (Fig 13). Biogeographic studies support *B*. *asper* as the first species complex to split from the *B*. *atrox* group in the Pliocene, around 3.02–2.32 Mya [90], and cladogenesis into lineages began soon thereafter, towards the end of the Pliocene [23]. The realization of the existence of large immunological conservation across *Bothrops* phylogeny emerged also from studies of the paraspecific effectiveness of the pentabothropic polyvalent antivenom SAB (*soro antibotrópico pentavalente*) produced by Instituto Butantan (São Paulo, Brazil) [52,76,79,91–94] using a pool of venoms from *B*. *jararaca* (50%), *B*. *jararacussu* (12.5%), *B*. *moojeni* (12.5%), *B*. *alternatus* (12.5%) and *B*. *neuwiedi* (12.5%) [95,96]. Further, an assessment of the ability of seven polyspecific antivenoms, produced in Argentina, Brazil, Perú, Bolivia, Colombia and Costa Rica using different immunization mixtures, to neutralize lethal, hemorrhagic, coagulant, defibrinogenating and myotoxic activities of the venoms of *B*. *diporus* (Argentina), *B*. *jararaca* (Brazil), *B*. *matogrossensis* (Bolivia), *B*. *atrox* (Perú and Colombia) and *B*. *asper* (Costa Rica) also showed a pattern of extensive cross-neutralization of all the venoms tested, with quantitative differences in the values of the effective doses of the antivenoms [93,97]. Similar results were obtained in a comparative a preclinical assessment of the efficacy of two whole IgG antivenoms, prepared in Perú and Costa Rica, to neutralize the most relevant toxic effects induced by the venoms of Peruvian *Bothrops atrox*, *B*. *brazili*, *B*. *barnetti* and *B*. *pictus* [98]. Both antivenoms were effective in the neutralization of these four venoms in a rodent model of envenoming, indicating an extensive immunological cross-reactivity exists between *Bothrops* spp. venoms from Perú and Costa Rica.

All the species complex groups within genus *Bothrops* include taxa that represent the main medically important venomous snakes in their range [9,99]. Our present results converge with previous studies in revealing the capacity of a number of bothropic antivenoms to neutralize, in preclinical tests, homologous and heterologous *Bothrops* venoms in Central and South America, and also highlight quantitative differences in their ED_50_s. The combination of antivenomics and *in vivo* neutralization assays provides relevant information to delineate the species spectrum and geographic range of clinical applicability of an antivenom. Now, the challenge is to conduct an extensive study to define the matrix of preclinical effectiveness of all the bothropic antivenoms against all the venoms of (medically relevant) species within the genus *Bothrops*.

## Supporting information

S1 Fig Antivenomics analysis of polyvalent antivenoms towards the venom of *B*. *asper* (*sensu stricto*). Panel **A** displays the fractionation by reverse-phase HPLC of the venom components. Proteins eluting in each peak (1–14) were assigned using the venomics information reported by Mora-Obando *et al*. [32]. Abbreviations for the venom components as in the legend of Fig 2A. Panels **B**-**G** represent RP-HPLC fractionations of the immunoretained fractions recovered in the affinity columns of immobilized antivenoms INS-COL (**B**), PROBIOL (**C**), ICP (**D**), INS-PERU (**E**), UCV (**F**), BIOL (**G**) incubated with increasing amounts of venom (100–1200 μg). Panels **H** and **I** display to chromatographic separations of the venom fraction retained in the mock matrix control and the naïve equine immunoglobulins control, respectively.
(TIF)

S2 Fig Antivenomics analysis of polyvalent antivenoms towards the venom of *B*. *rhombeatus*. Panel **A** displays the fractionation by reverse-phase HPLC of the venom components. Proteins eluting in each peak (1–14) were assigned using the venomics information reported by Mora-Obando *et al*. [32]. Abbreviations for the venom components as in the legend of Fig 3A. Panels **B**-**G** represent RP-HPLC fractionations of the immunoretained fractions recovered in the affinity columns of immobilized antivenoms INS-COL (**B**), PROBIOL (**C**), ICP (**D**), INS-PERU (**E**), UCV (**F**), BIOL (**G**) incubated with increasing amounts of venom (100–1200 μg). Panels **H** and **I** display to chromatographic separations of the venom fraction retained in the mock matrix control and the naïve equine immunoglobulins control, respectively.
(TIF)

S3 Fig Immunocapture capacity of polyvalent antivenoms towards the venom of *B*. *ayerbei*. Panel **A** displays the fractionation by reverse-phase HPLC of the venom components. Proteins eluting in each peak (1–14) were assigned using the venomics information reported by Mora-Obando *et al*. [28]. Abbreviations for the venom components as in the legend of Fig 4A. Panels **B**-**G** represent RP-HPLC fractionations of the immunoretained fractions recovered in the affinity columns of immobilized antivenoms INS-COL (**B**), PROBIOL (**C**), ICP (**D**), INS-PERU (**E**), UCV (**F**), BIOL (**G**) incubated with increasing amounts of venom (100–1200 μg). Panels **H** and **I** display to chromatographic separations of the venom fraction retained in the mock matrix control and the naïve equine immunoglobulins control, respectively.
(TIF)

S1 Table MS/MS assignment of protein bands excised from SDS-PAGE analysis of the six polyvalent antivenoms studied. (XLSX)

S2 Table Concentration-dependent immunoretained (RET) *B*. *asper* (Cauca) venom proteins by INS-COL antivenom affinity column. Maximal binding for each RP-HPLC fraction is highlighted in yellow background. Green background, amount (in μg) of toxin family proteins immunoretained in the affinity columns.
(XLSX)

S3 Table Concentration-dependent immunoretained (RET) *B*. *asper* (Cauca) venom proteins by PROBIOL antivenom affinity column. Maximal binding for each RP-HPLC fraction is highlighted in yellow background. Green background, amount (in μg) of toxin family proteins immunoretained in the affinity columns.
(XLSX)

S4 Table Concentration-dependent immunoretained (RET) *B*. *asper* (Cauca) venom proteins by ICP antivenom affinity column. Maximal binding for each RP-HPLC fraction is highlighted in yellow background. Green background, amount (in μg) of toxin family proteins immunoretained in the affinity columns.
(XLSX)

S5 Table Concentration-dependent immunoretained (RET) *B*. *asper* (Cauca) venom proteins by INS-PERU antivenom affinity column. Maximal binding for each RP-HPLC fraction is highlighted in yellow background. Green background, amount (in μg) of toxin family proteins immunoretained in the affinity columns.
(XLSX)

S6 Table Concentration-dependent immunoretained (RET) *B*. *asper* (Cauca) venom proteins by UCV antivenom affinity column. Maximal binding for each RP-HPLC fraction is highlighted in yellow background. Green background, amount (in μg) of toxin family proteins immunoretained in the affinity columns.
(XLSX)

S7 Table Concentration-dependent immunoretained (RET) *B*. *asper* (Cauca) venom proteins by BIOL antivenom affinity column. Maximal binding for each RP-HPLC fraction is highlighted in yellow background. Green background, amount (in μg) of toxin family proteins immunoretained in the affinity columns.
(XLSX)

S8 Table Concentration-dependent immunoretained (RET) *B*. *rhombeatus* (Cauca) venom proteins by INS-COL antivenom affinity column. Maximal binding for each RP-HPLC fraction is highlighted in yellow background. Green background, amount (in μg) of toxin family proteins immunoretained in the affinity columns.
(XLSX)

S9 Table Concentration-dependent immunoretained (RET) *B*. *rhombeatus* (Cauca) venom proteins by PROBIOL antivenom affinity column. Maximal binding for each RP-HPLC fraction is highlighted in yellow background. Green background, amount (in μg) of toxin family proteins immunoretained in the affinity columns.
(XLSX)

S10 Table Concentration-dependent immunoretained (RET) *B*. *rhombeatus* (Cauca) venom proteins by ICP antivenom affinity column. Maximal binding for each RP-HPLC fraction is highlighted in yellow background. Green background, amount (in μg) of toxin family proteins immunoretained in the affinity columns.
(XLSX)

S11 Table Concentration-dependent immunoretained (RET) *B*. *rhombeatus* (Cauca) venom proteins by INS-PERU antivenom affinity column. Maximal binding for each RP-HPLC fraction is highlighted in yellow background. Green background, amount (in μg) of toxin family proteins immunoretained in the affinity columns.
(XLSX)

S12 Table Concentration-dependent immunoretained (RET) *B*. *rhombeatus* (Cauca) venom proteins by UCV antivenom affinity column. Maximal binding for each RP-HPLC fraction is highlighted in yellow background. Green background, amount (in μg) of toxin family proteins immunoretained in the affinity columns.
(XLSX)

S13 Table Concentration-dependent immunoretained (RET) *B*. *rhombeatus* (Cauca) venom proteins by BIOL antivenom affinity column. Maximal binding for each RP-HPLC fraction is highlighted in yellow background. Green background, amount (in μg) of toxin family proteins immunoretained in the affinity columns.
(XLSX)

S14 Table Concentration-dependent immunoretained (RET) *B*. *ayerbei* (Cauca) venom proteins by INS-COL antivenom affinity column. Maximal binding for each RP-HPLC fraction is highlighted in yellow background. Green background, amount (in μg) of toxin family proteins immunoretained in the affinity columns.
(XLSX)

S15 Table Concentration-dependent immunoretained (RET) *B*. *ayerbei* (Cauca) venom proteins by PROBIOL antivenom affinity column. Maximal binding for each RP-HPLC fraction is highlighted in yellow background. Green background, amount (in μg) of toxin family proteins immunoretained in the affinity columns.
(XLSX)

S16 Table Concentration-dependent immunoretained (RET) *B*. *ayerbei* (Cauca) venom proteins by ICP antivenom affinity column. Maximal binding for each RP-HPLC fraction is highlighted in yellow background. Green background, amount (in μg) of toxin family proteins immunoretained in the affinity columns.
(XLSX)

S17 Table Concentration-dependent immunoretained (RET) *B*. *ayerbei* (Cauca) venom proteins by INS-PERU antivenom affinity column. Maximal binding for each RP-HPLC fraction is highlighted in yellow background. Green background, amount (in μg) of toxin family proteins immunoretained in the affinity columns.
(XLSX)

S18 Table Concentration-dependent immunoretained (RET) *B*. *ayerbei* (Cauca) venom proteins by UCV antivenom affinity column. Maximal binding for each RP-HPLC fraction is highlighted in yellow background. Green background, amount (in μg) of toxin family proteins immunoretained in the affinity columns.
(XLSX)

S19 Table Concentration-dependent immunoretained (RET) *B*. *ayerbei* (Cauca) venom proteins by BIOL antivenom affinity column. Maximal binding for each RP-HPLC fraction is highlighted in yellow background. Green background, amount (in μg) of toxin family proteins immunoretained in the affinity columns.
(XLSX)

S20 Table Neutralization of biological activities of south-western Colombian *B*. *asper* lineage venoms by polyvalent antivenoms (values adjusted according to protein concentration per vial). (XLSX)
