# Supplementary figures and images for: Antivenomics and in vivo preclinical efficacy of six Latin American antivenoms towards south-western Colombian Bothrops asper lineage venoms
Source: PLoS Negl Trop Dis. 2021 Feb 1;15(2):e0009073. doi: 10.1371/journal.pntd.0009073 (PMC7877754; doi:10.1371/journal.pntd.0009073)

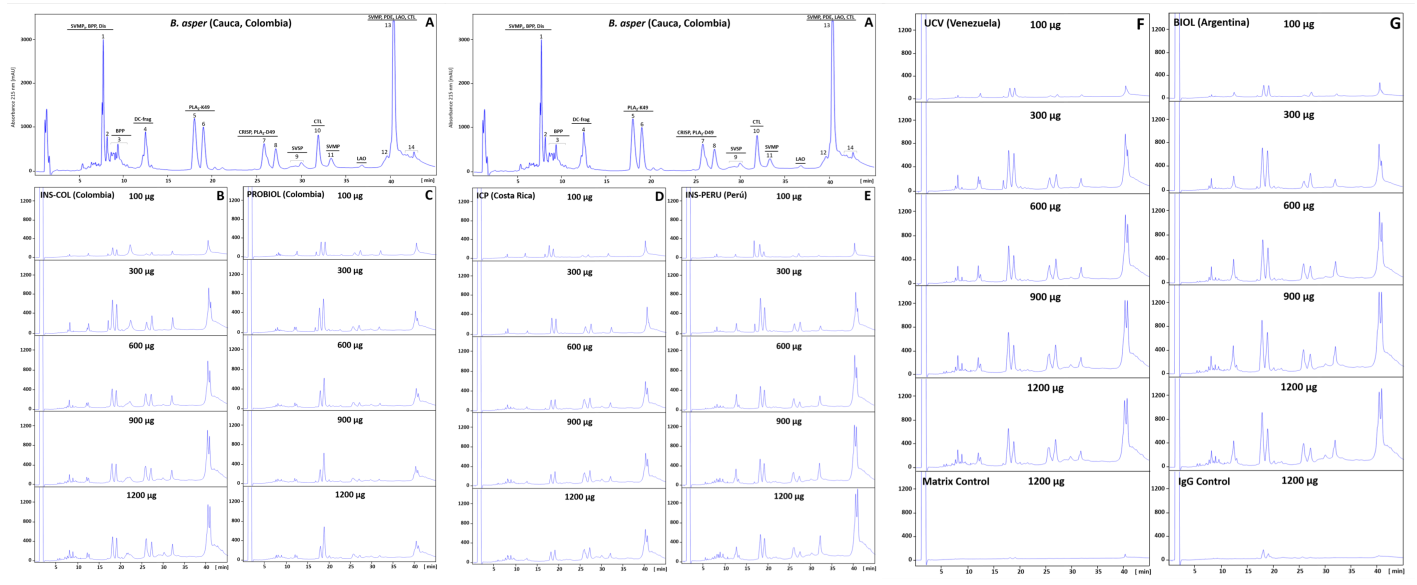

Supplement: S1 Fig — Panel A displays the fractionation by reverse-phase HPLC of the venom components. Proteins eluting in each peak (1–14) were assigned using the venomics information reported by Mora-Obando et al. [32]. Abbreviations for the venom components as in the legend of Fig 2A. Panels B-G represent RP-HPLC fractionations of the immunoretained fractions recovered in the affinity columns of immobilized antivenoms INS-COL (B), PROBIOL (C), ICP (D), INS-PERU (E), UCV (F), BIOL (G) incubated with increasing amounts of venom (100–1200 μg). Panels H and I display to chromatographic separations of the venom fraction retained in the mock matrix control and the naïve equine immunoglobulins control, respectively. (TIF) [file pntd.0009073.s001.tif]

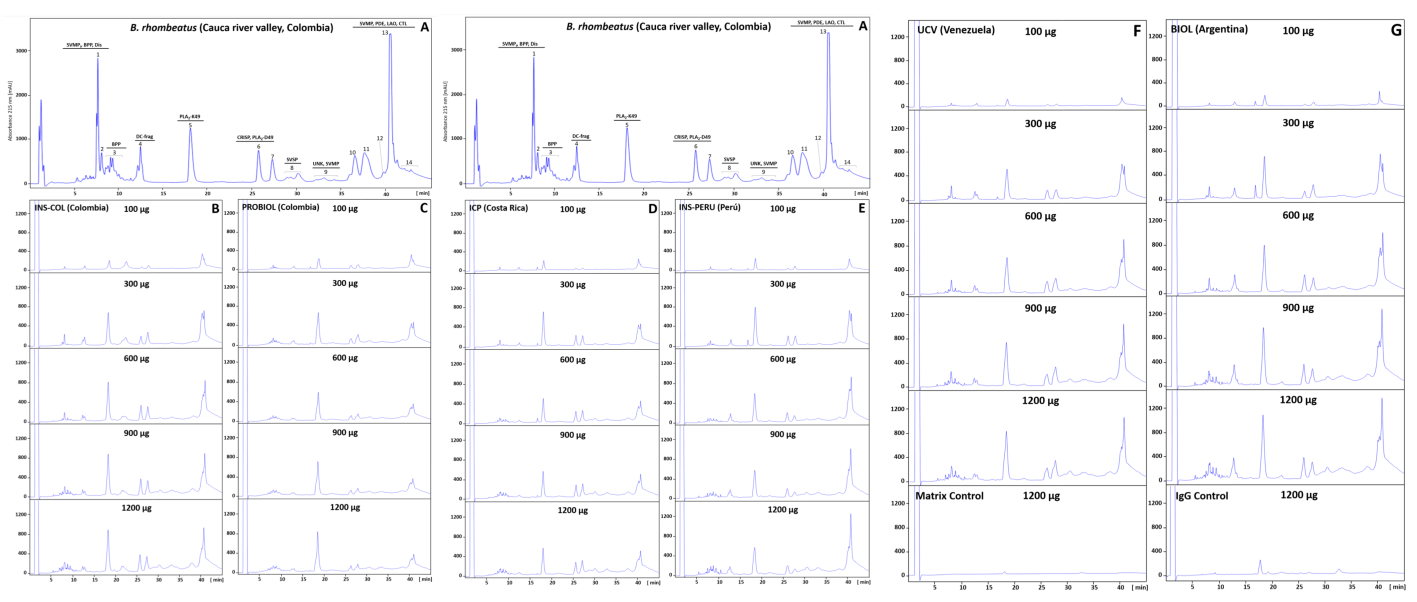

Supplement: S2 Fig — Panel A displays the fractionation by reverse-phase HPLC of the venom components. Proteins eluting in each peak (1–14) were assigned using the venomics information reported by Mora-Obando et al. [32]. Abbreviations for the venom components as in the legend of Fig 3A. Panels B-G represent RP-HPLC fractionations of the immunoretained fractions recovered in the affinity columns of immobilized antivenoms INS-COL (B), PROBIOL (C), ICP (D), INS-PERU (E), UCV (F), BIOL (G) incubated with increasing amounts of venom (100–1200 μg). Panels H and I display to chromatographic separations of the venom fraction retained in the mock matrix control and the naïve equine immunoglobulins control, respectively. (TIF) [file pntd.0009073.s002.tif]

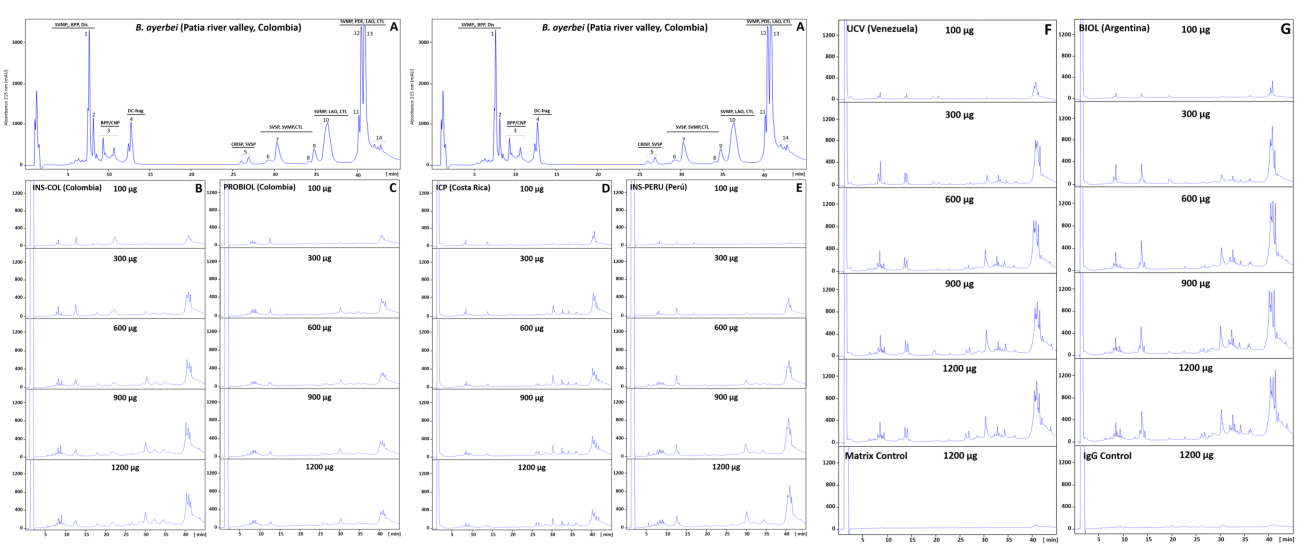

Supplement: S3 Fig — Panel A displays the fractionation by reverse-phase HPLC of the venom components. Proteins eluting in each peak (1–14) were assigned using the venomics information reported by Mora-Obando et al. [28]. Abbreviations for the venom components as in the legend of Fig 4A. Panels B-G represent RP-HPLC fractionations of the immunoretained fractions recovered in the affinity columns of immobilized antivenoms INS-COL (B), PROBIOL (C), ICP (D), INS-PERU (E), UCV (F), BIOL (G) incubated with increasing amounts of venom (100–1200 μg). Panels H and I display to chromatographic separations of the venom fraction retained in the mock matrix control and the naïve equine immunoglobulins control, respectively. (TIF) [file pntd.0009073.s003.tif]
